# A prototype-augmented graph representation learning framework for identifying brain disorder-associated genes and facilitating drug repurposing

**DOI:** 10.1371/journal.pcbi.1014323

**Published:** 2026-05-29

**Authors:** Jiafang Li, Yifei Li, Siying Lin, Jiahua Rao, Huiying Zhao

**Affiliations:** 1 Department of Medical Research Center, Sun Yat-Sen Memorial Hospital, Sun Yat-Sen University, Guangzhou, Guangdong, China; 2 Guangdong Provincial Key Laboratory of Malignant Tumor Epigenetics and Gene Regulation, Guangzhou, Guangdong, China; 3 School of Computer Science and Engineering, Sun Yat-sen University, Guangzhou, Guangdong, China; Shanghai Institute of Nutrition and Health, Chinese Academy of Sciences, CHINA

## Abstract

Many genetic loci were identified as associated with neuropsychiatric disorders and neurodegenerative disorders by Genome-wide association studies (GWAS). How these loci impact these diseases is unclear. Advances in deep-learning approaches and multi-omics data have the potential to link GWAS findings with disease mechanisms. Here, we proposed the Multi-omics Graph Transformer Network (MOGT), a semi-supervised graph neural network that leverages graph representation learning to model biological networks derived from multi-omics data to predict disease-associated genes. MOGT outperforms the current approaches in disease gene prediction for two psychiatric disorders and three neurodegenerative/neurological diseases. High-risk genes (HRGs) for Parkinson’s disease (PD) predicted by MOGT were used to drug discovery by integrating with the CMAP database. Finally, 10 drugs were identified as potential candidates. Among them, the effect of drug UK-356618 was experimentally verified in a primary neuron model, showing that UK-356618 reversed the abnormal expression of PD-associated genes and improved the cell-level phenotypes of PD. Together, these results indicate that MOGT can be used to identify HRGs for brain disorders, and these predicted HRGs provide high-level insights into the mechanisms and treatments of brain disorders.

## Author summary

Genes that contribute to brain disorders, such as Schizophrenia, Alzheimer’s disease, and Parkinson’s disease, often arise from many small changes across the genome, making it difficult to pinpoint which genes truly play important roles. Although large amounts of genomic data are now available, each type of data captures only part of the picture. Although many studies have used advanced computational models to identify disease-related genes, these methods are designed for cancers. To address this problem, we proposed a framework named MOGT, leveraging graph representation learning to model biological networks derived from multi-omics data, which allows us to merge diverse genomic signals for detecting genes that are likely to influence brain-related conditions. By applying MOGT across multiple brain disorders, we were able to identify meaningful genes that often play central roles in brain disorders development. Notably, we also show that the high-risk genes predicted by MOGT can be used to facilitate novel drug candidates. Overall, MOGT can be used to identify high-risk genes for brain disorders, and these predicted high-risk genes provide high-level insights into the mechanisms and treatments of brain disorders.

## Introduction

Genome-wide association study (GWAS) provides new insights into the genetic basis of complex traits and diseases [1]. Most of the significant variants identified by GWAS are located in introns or intergenic regions, making it hard to link their functions with genes, which has impeded the applications of GWAS in improving the understanding of disease mechanisms and in drug discovery.

Researchers have made great efforts to identify disease risk genes by integrating GWAS loci and multi-omics data. Fan et al. integrated GWAS data with transcriptomic information to identify genes associated with Alzheimer’s disease [2]. OSCA is another commonly used method for detecting associations between genes and complex traits by leveraging omics data [3]. iRIGS, an unsupervised Bayesian framework, integrated multi-omics data and a generic gene-gene network constructed from Gene Ontology (GO) to identify Schizophrenia (SCZ) risk genes [4]. He et al. employed a Bayesian framework (iGOAT) to integrate risk single nucleotide polymorphism(SNPs) with long-range chromatin interactions to predict high-risk genes and achieve better prediction [5]. All these methods are unsupervised learning, which lack a ground truth evaluation and are limited to dealing with high-dimensional features.

However, disease-associated SNPs account only for a fraction of the investigated traits’ heritability [6,7]. Several studies have developed epistasis interactions between SNPs detection tools, including NeEDL [8], LINDEN [9], MACOED [10], and BiologicalEpistasis [11] in an attempt to explain the heritability of missing SNPs through epistatic SNP interactions, which are collectively, rather than individually, associated with the phenotype. More broadly, prior work has analyzed the topological and statistical properties of epistatic SNP–SNP networks, highlighting their potential as structured representations of genetic interactions and suggesting graph-based learning as a natural modeling direction. However, existing SNP–SNP network methods primarily focus on variant-level epistasis detection or feature selection, treating SNP–SNP interactions as the final object of analysis.

Recently, Graph Neural Network (GNN) has been used to incorporate graph structure into a deep learning framework for representing biological information graphs. In general, the representations of nodes in the same category are relatively similar in linking prediction tasks. Seo et al. proposed PGIB, which incorporates prototype learning within the information bottleneck framework to provide prototypes for the key subgraph from the input graph to improve accuracy in linking prediction tasks [12]. Many researchers have introduced GNN into the task of identifying disease risk genes, such as EMOGI [13], CGmega [14], and TREE [15]. EMOGI leverages Graph Convolutional Networks (GCNs) to integrate protein-protein interaction (PPI) networks and multi-omics pan-cancer data, including copy number changes, DNA methylation, and gene expression, to find novel cancer genes. CGMega utilized a transformer-based graph attention neural network to combine the PPI network and multi-omics data and fine-tuned it on specific cancers to predict cancer genes. TREE leverages graph representation learning and the integration of multi-omics data with the topologies of homogeneous and heterogeneous networks of biological interactions to predict cancer genes. Most of the deep learning-based approaches are used in predicting cancer genes and are not often utilized in GWAS studies for detecting genes associated with neuropsychiatric disorders or neurodegenerative disorders.

Indeed, there are a large number of GWAS performed to identify genetic factors associated with neuropsychiatric diseases, such as SCZ and Bipolar disorder (BP). GWAS for SCZ has identified more than 200 significant loci, which were summarized in the GWAS catalog [16,17]. Moreover, GWAS for neurodegenerative diseases, such as Alzheimer’s disease (AD) and Parkinson’s disease (PD), have discovered many significant loci to characterize the diseases [18,19]. Linking these genetic loci to risk genes by the deep-learning model is important for discovering novel mechanisms and medicines. Genes affected by trait-associated variation may provide opportunities for drug repurposing [20]. In fact, previous research has shown that drug repurposing aims to identify new uses for approved or investigational drugs that are outside the scope of the original medical indication, with relatively low cost and fewer safety concerns [21,22]. Several studies have developed computational frameworks to explore drug repurposing, including iGOLD [22], a network-based artificial intelligence framework [23]. These methods prove the utility of network-based methodologies for accelerating target identification and therapeutic discovery.

In this study, we proposed the Multi-omics Graph Transformer Network (MOGT), a semi-supervised graph neural network that integrates the multi-omics data of genes and the topology information of biological networks. We collected SNPs associated with brain disorders. Candidate genes were defined as those within 1Mb of index SNPs. To construct the multi-omics representation graph, we represented genes as nodes and SNP-SNP interactions as edges. The node features were the multi-omics data of genes, including differential expression (DE), enhancer-promoter interactions (EPI) in patients and controls, and gene expression in five brain regions (Parietal Lobe, Frontal Lobe, Temporal Lobe, Cerebellum, and occipital Lobe) of adolescents and adults. SNPs in the SNP-SNP interaction networks are characterized by the associations between genotypes and phenotypes. The novelty of our study in using SNP-SNP interaction lies in two respects. First, methods such as NeEDL [8] and other epistasis-focused SNP-network approaches [9–11] primarily aim to identify interacting SNP sets or detect epistatic effects at the variant level, often in a feature-selection or discovery setting. In contrast, our method uses SNP–SNP interactions as a latent relational structure to inform gene-level disease risk prediction, which is the ultimate biological and clinical objective of our study. Secondly, Prior epistasis network methods typically treat SNP–SNP interactions as the final object of analysis. In our framework, SNP–SNP interactions are instead used to shape message passing and information flow within a graph-based model, acting as a structural prior that constrains how genetic signals propagate across variants and aggregate to genes. This shifts the role of SNP–SNP interactions from discovery targets to inductive bias. For four brain disorders (SCZ, AD, PD, and BP), MOGT achieved the average Area Under the Precision-Recall Curve (AUPRC) above 0.67, outperforming state-of-the-art (SOTA) models, indicating strong predictive performance and robustness. We applied MOGT to two psychiatric disorders, SCZ and BP, and three neurodegenerative/neurological diseases, AD, PD, and Migraine (MG), to predict disease-associated genes. The HRGs predicted by MOGT are used to discover drug candidates for PD and found 10 potential new drugs. The top-ranked and fifth-ranked drugs, UK-356618 and GSK-1059615, were validated by both the PD primary neuron model and primary astrocyte model. The result indicated that the drug UK-356618 has shown effectiveness in reversing the expression of PD-associated genes and improving the phosphorylation of the protein α-SYN associated with the PD phenotype. Together, these results suggest that MOGT can be used to discover novel disease-associated genes and provide new insights into the treatment of brain disorders.

## Results

### Overview of MOGT architecture

**Fig 1:**
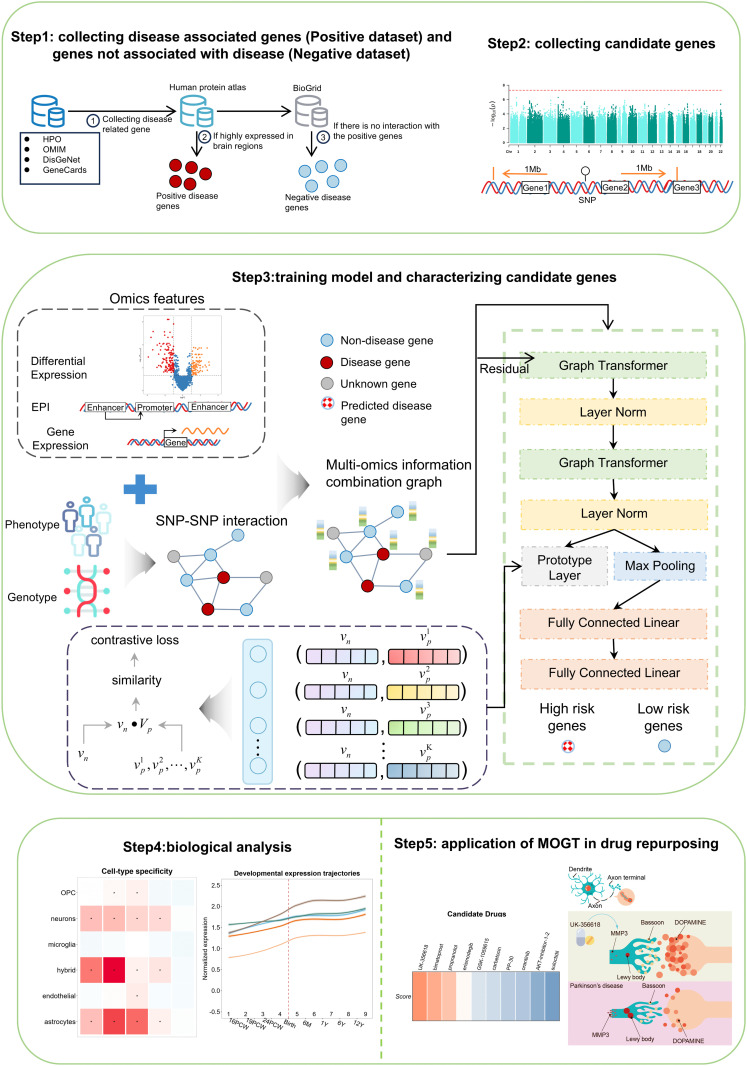
Overview of MOGT framework. First, we collected positive and negative samples and candidate genes from different sources. To combine multi-omics information, we created a graph where nodes represented genes and edges were derived from SNP-SNP interactions. Node features were composed of the multi-omics features of genes, including differential expression (DE), enhancer-promoter interactions (EPI) in patients and controls, and gene expression in five brain regions in adolescents and adults. Next, a prototype-based Graph Transformer was constructed. The biological features of HRGs were explored and compared with those of LRGs. Finally, MOGT was applied to drug repurposing.

We proposed a new framework, MOGT, for detecting risk genes of brain disorders by graph transformer and Prototype Learning. The overall framework of MOGT and the application of MOGT are presented in Fig 1. First, we established positive and negative datasets for training the model (Methods). According to the GWAS summary data, we generate candidate genes based on the distances of significant SNPs to genes. Secondly, MOGT is constructed by integrating multi-omics data, including transcriptomics and epigenomics, with SNP-SNP interactions. We constructed a multi-omics information graph, in which the nodes represent genes and the edges are interactions between SNPs observed in patients and controls from the UK-Biobank (Methods). To capture the most significant patterns of the risk genes, MOGT introduces prototype learning. The output of MOGT is the probability of each gene associated with a given brain disorder. The ability of MOGT in predicting risk genes is tested in the hold-out test and evaluated by the area under the receiver operating characteristic curve (AUROC), F1-score, and AUPRC. Thirdly, MOGT was applied to predict the risk genes for SCZ, BP, AD, PD, and MG, respectively. The HRGs (High-risk genes) and the LRGs (Low-risk genes) were compared on their biological functions involving brain disorders. Finally, the HRGs predicted by MOGT were used for drug repurposing through constructing gene co-expression networks based on WGCNA [24] and Diffcoex [25]. The most significant gene co-expression module is integrated with the CMAP database. The application in PD identified 10 drug candidates. Among them, UK-356618 and GSK-1059615 were validated experimentally by the primary neuron model and the primary astrocyte model, respectively. The result indicated UK-356618 having the ability to reverse the abnormal expression of MMP3 and BSN in a PD primary neuron model and subsequently rescued PD disease-relevant cellular markers. BSN is predicted as HRG of PD by MOGT.

### MOGT is effective in predicting brain disorder genes

**Fig 2:**
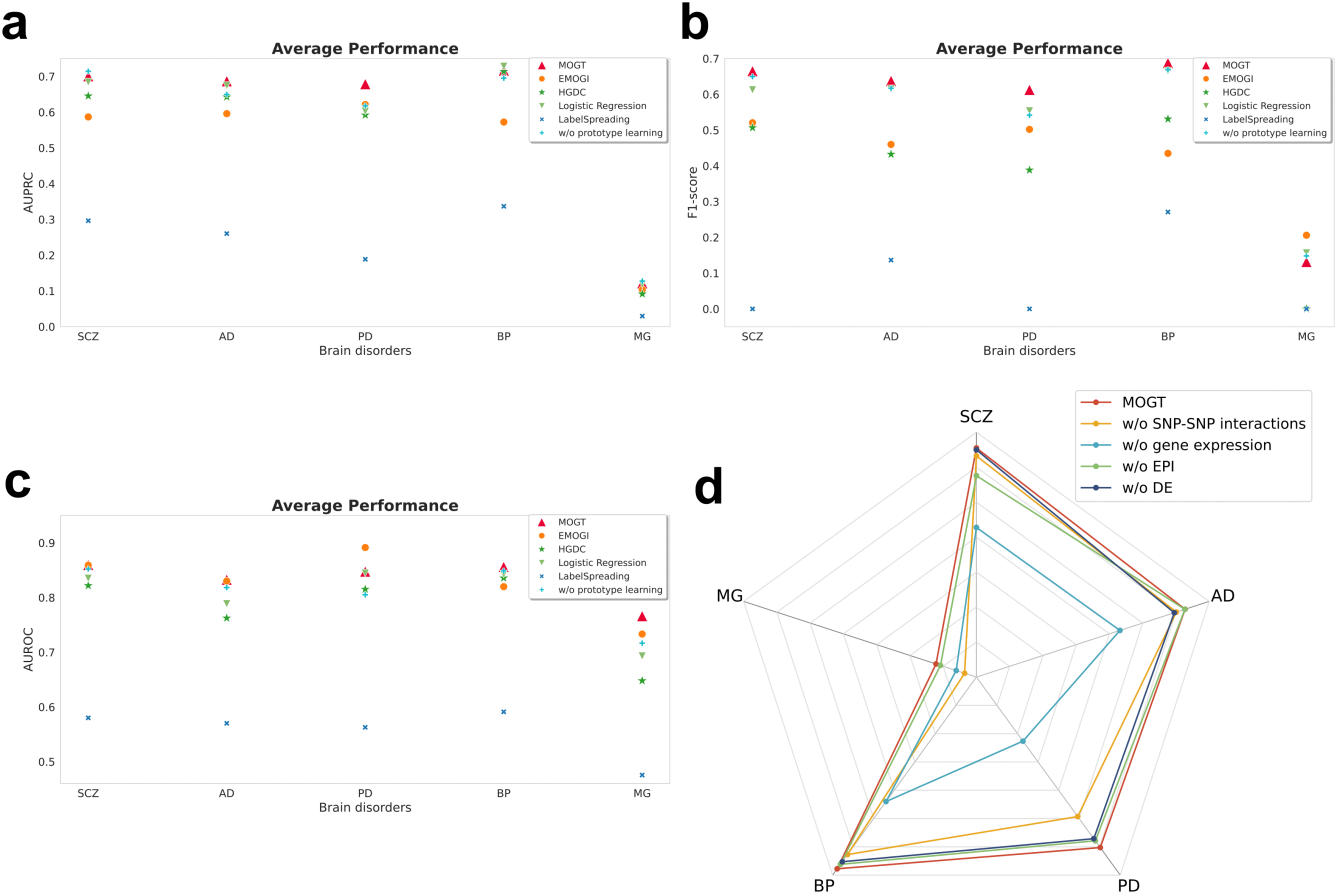
Performance of MOGT in predicting high-risk genes for five brain disorders, SCZ, AD, PD, BP, and MG. **(a-c)**: Methods comparison on SCZ, AD, PD, BP, and MG. Performance was evaluated on the hold-out test set using AUROC, F1-score, and AUPRC, with model selection conducted via 5-fold cross-validation. **(d)**: F1-score performance of MOGT in the ablation study, evaluated on the hold-out test set with model selection via 5-fold cross-validation.

MOGT was applied to two psychiatric disorders, SCZ and BP, and three neurodegenerative/neurological diseases, AD, PD, and MG. The performance of MOGT in predicting risk genes was evaluated by the hold-out test set (see “Methods”). In the predicting risk genes task, MOGT achieved AUROC of 0.8596, 0.8327, 0.8475, 0.8561, and 0.7661, and the area under the precision-recall curve (AUPRC) of 0.7007, 0.6867, 0.6786, 0.7165, and 0.1216 for SCZ, AD, PD, BP, and MG, respectively (Fig 2a-2c, S1, S2 Tables) in the hold-out test set.

To demonstrate the advances of MOGT in the brain disorder genes prediction task, we compared MOGT with specific models designed for gene classification, encompassing EMOGI and HGDC, feature-only baseline logistic regression, and graph-only baseline LabelSpreading (see “Methods”). All models were evaluated using the same input features and networks. By computing AUPRC, AUROC, and F1-score, MOGT outperformed all other methods (Fig 2a-2c). The EMOGI has achieved AUPRC 0.5867, 0.5959, 0.6214, 0.5724, and 0.1039 for predicting high-risk genes of SCZ, AD, PD, BP, and MG, respectively, while the HGDC has achieved AUPRC 0.6453, 0.6426, 0.5919, 0.7138, and 0.0916 for predicting high-risk genes of SCZ, AD, PD, BP, and MG, respectively. The performances of these four methods in AUROC and F1-score are shown in Fig 2b-2c and S2 Table. The result indicated that MOGT has superior performance compared to these two methods. We performed an ablation test to examine the effects of prototype learning (Fig 2a-2c, S2 Table). The result indicated that prototype learning makes a significant contribution to improving the performance of MOGT. We also reported the number of positive and negative samples in the test set for each disorder (S3 Table). We further noted that the test set for MG contained 24 positive and 826 negative samples, for which the random baseline AUPRC under this prevalence was approximately 0.0282. Although the absolute AUPRC for MG (AUPRC = 0.1216) is lower than for other disorders, it still represented about a 4.3-fold improvement over the random baseline. In fact, AUPRC values cannot be directly compared across tasks with markedly different class prevalences, and the lower absolute AUPRC observed for MG is largely driven by its extreme class imbalance and smaller sample size rather than a failure of the model. However, MG remained substantially lower than those observed for other diseases. We noted that the class imbalance differs markedly across diseases, with MG exhibiting a much higher negative-to-positive sample ratio compared to other diseases (MG: 35:1; PD: 5.29:1), which may partly contribute to the observed performance differences. In fact, severe class imbalance may bias the model toward the majority class, leading to conservative predictions and reduced sensitivity for identifying positive samples, particularly in gene prioritization tasks where true positive labels are sparse and incomplete. Overall, MOGT outperforms competing methods on most disease–metric combinations and demonstrates consistently strong performance across different diseases and evaluation metrics.

We performed an ablation test to examine the effects of SNP-SNP interactions in the prediction. The result indicated that MOGT without using SNP-SNP interactions achieved an average F1-score of 0.6423, which is lower than the F1-score of 0.6649 achieved by the MOGT model with SNP-SNP interactions. This result proved that SNP-SNP interactions provide a consistent but modest performance gain. Here, we kept the top 1% of SNP-SNP interactions detected from the UK-Biobank instead of all interactions in the prediction because the increase in the number of SNP-SNP interactions used in the MOGT has not brought a dramatic increase in the F1-score performance (S1 Fig).

In the ablation test, the exclusion of gene expression features results in the decrease of MOGT performance from F1-score 0.6649 to 0.4377 in SCZ, 0.6876 to 0.4499 in BP, 0.6376 to 0.4417 in AD, 0.6126 to 0.2365 in PD, and 0.1315 to 0.0708 in MG (Figs 2d, S2, S4 Table). The ablation test was further performed to evaluate the effects of the other features in the prediction. All the results indicated that gene expression features contribute the most.

To assess the robustness of gene prioritization with respect to GWAS-derived SNP-to-gene mapping choices, we conducted a sensitivity analysis by comparing two commonly used window sizes (±500 Kb and ±1 Mb) for defining candidate genes. Notably, in our dataset, the ± 500 Kb candidate gene set is fully contained within the ± 1 Mb window, allowing us to evaluate whether expanding the candidate gene space alters downstream prioritization outcomes. As shown in S5 Table, gene-level risk assignments are highly consistent between the two settings. For four diseases (SCZ, AD, BP, and PD), approximately 99% of genes retain identical risk classifications (high-risk or low-risk) under both window definitions. For MG, a lower but still substantial agreement (83.3%) was observed, likely reflecting increased variability due to the smaller sample size. These results indicated that, within this range, the choice of SNP-to-gene mapping window size has a limited impact on the final prioritization results. While window size influences the initial candidate gene space, the downstream model predictions remain largely stable, supporting the robustness of our framework to this key GWAS-related design choice.

To evaluate the robustness of our model against negative sampling strategies, we conducted an additional sensitivity analysis by constructing an alternative negative set that does not exclude interactions from the BioGRID database. Under this more challenging setting, the AUPRC decreased by an average of 9.4% while AUROC exhibited a comparatively smaller decrease of 3.7% (S6 Table). This performance degradation is expected, as including BioGRID interactors in the negative set introduces harder negatives and increases label ambiguity. Specifically, some negative samples may be topologically or functionally similar to positive pairs, or may correspond to yet-unlabeled true interactions, thereby making the classification task substantially more difficult. The relatively smaller decline in AUROC compared to AUPRC suggested that the model’s ranking capability is largely preserved, while precision is more sensitive to the increased ambiguity in the negative class. Overall, these results demonstrated that the proposed method is not solely dependent on topology-driven negative sampling, and its predictive ability remains robust under alternative and more challenging negative sampling schemes.

### Applications of MOGT in multiple brain disorders

Applications of MOGT have been performed using GWAS summary data of SCZ, BP, AD, PD, and MG (Methods). MOGT identified 201, 117, 242, 81, and 67 genes as HRGs for SCZ, BP, AD, PD, and MG, respectively. Simultaneously, MOGT has identified 1,187, 353, 872, 519, and 147 LRGs for SCZ, BP, AD, PD, and MG, respectively (S7 Table).

**Fig 3:**
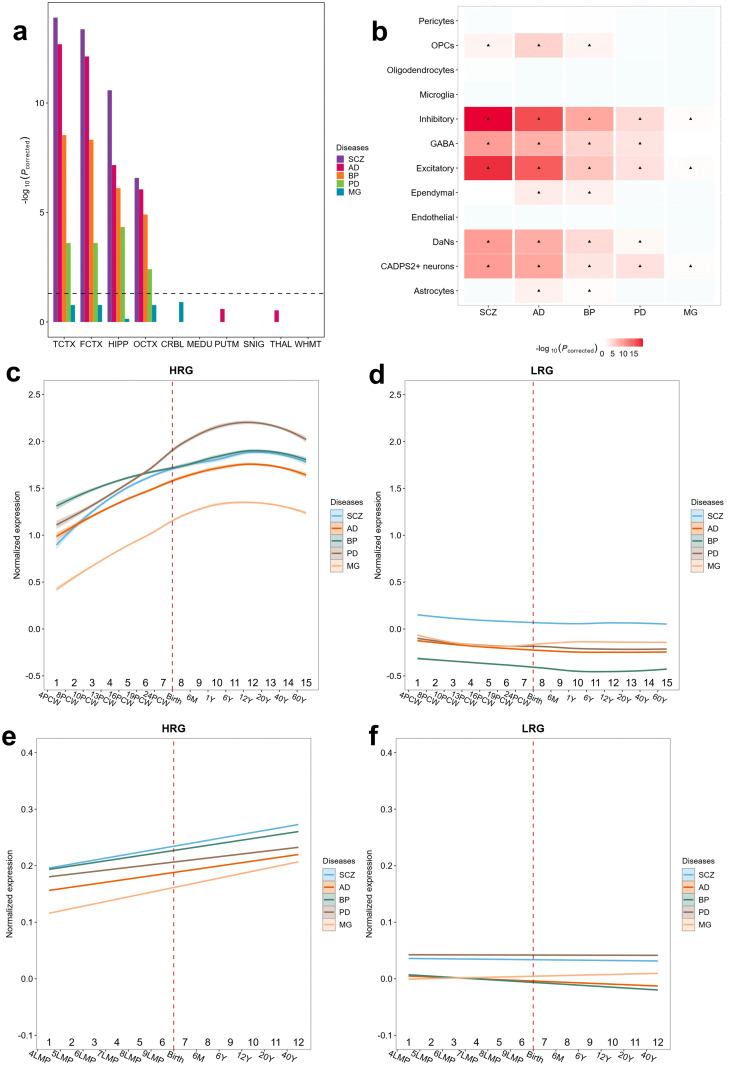
Analysis of the expression of HRGs. **(a)** Tissue specificity analysis of HRGs compared to LRGs using expression profiles from BrainEAC (Methods). **(b)** Cell specificity analysis of HRGs compared to LRGs (Methods). “▴” denotes significant result (*p*_*corrected*_ < 0.05, Wilcoxon test, with BH(Benjamini-Hochberg) correction. **(c, d)** Developmental expression trajectories of the HRGs and LRGs during the entire brain developmental stages. The dashed line represents birth. The shaded areas represent the 95% confidence interval of expression levels. **(e, f)** Developmental expression trajectories of the HRGs and LRGs during the entire brain developmental stages in astrocyte and neural cells. The dashed line represents birth. The shaded areas represent the 95% confidence interval of expression levels.

The expression specificity of HRGs and LRGs in tissues was evaluated by a tissue specificity analysis [26]. The expression data of HRGs and LRGs are from the Genotype-Tissue Expression (GTEx) project (Methods). The result indicated that HRGs exhibited greater tissue specificity in the brain tissues than LRGs (S3 Fig). In particular, we observed that HRGs for AD and BP were highly specifically expressed in the left ventricle (LV) (the multiple-testing adjusted p-value, *p*_*corrected*_ < 2.3653 × 10^−3^, Wilcoxon test with BH correction) and atrial appendage (*p*_*corrected*_ < 6.5543 × 10^−3^, Wilcoxon test with BH correction) than LRGs, and AD and SCZ were highly specifically expressed in the pituitary (*p*_*corrected*_ < 2.9574 × 10^−4^, Wilcoxon test with BH correction) than LRGs. The tissue specificity of HRGs in the brain was further evaluated by using expression data from BrainEAC, which indicated HRGs associated with all four brain disorders (SCZ, AD, BP, and PD) exhibited significant tissue specificity (*p*_*corrected*_<0.05, Wilcoxon test with FDR correction) in the temporal cortex (TCTX), frontal cortex (FCTX), hippocampus (HIPP), and occipital cortex (OCTX) compared to the LRGs (Fig 3a).

The cell-type specificity analysis is to identify the cell types enriched by the HRGs and LRGs [27]. The result indicated that the HRGs associated with SCZ, BP, AD, and PD have more pronounced cell-type specificity in neurons (*p*_*corrected*_ < 0.001, Wilcoxon test with BH correction), astrocytes (*p*_*corrected*_ < 0.0135, Wilcoxon test with BH correction), and hybrid (*p*_*corrected*_ < 0.0119, Wilcoxon test with BH correction) than the LRGs. Furthermore, the HRGs associated with SCZ, AD, and BP have more cell-type specificity in oligodendrocytes (*p*_*corrected*_ < 0.0078, Wilcoxon test with BH correction) than the LRGs (S4 Fig). We performed cell-specific analysis with single-cell transcriptomics of midbrain tissue, and found that the HRGs associated with SCZ, BP, AD, and PD have more pronounced cell-type specificity in neuronal cells, including GABAergic, Inhibitory, Excitatory, Dopaminergic neurons (DaNs), and CADPS2 overexpression neuronal cells than the LRGs (Fig 3b).

The analysis of HRGs expression levels in different brain developmental stages indicated that the HRGs associated with the five brain disorders showed remarkably similar expression patterns with increases during stages 1–11 (4PCW ≤ age < 12Y), slight decreases at stages 13–15 (adult) and a peak at stage 12 (12Y ≤ age < 20Y, adolescence) while this pattern was absent for LRGs. We noted that the HRGs associated with brain disorders exhibited higher expression levels than the LRGs during brain development (Fig 3c, 3d). During brain development, the HRGs associated with four brain disorders (SCZ, AD, BP, and PD) that exhibited pronounced cell-type specificity in neurons and astrocytes (S4 Fig) exhibit similar expression patterns in these two cell types (Figs 3e, 3f; S5, S6).

In this study, downstream analyses are designed not as absolute enrichment tests, but as relative comparisons between HRGs and LRGs derived from the same model, with the aim of validating whether genes prioritized by the model show stronger disease relevance than low-confidence genes. Introducing matched gene controls would fundamentally alter this comparison framework, as matched non-HRG genes neither represent low-risk model outputs nor preserve the ranking structure learned by the model, making many of the downstream analyses (cell-type specificity, tissue relevance, and developmental profiling) difficult to interpret in a consistent manner. To address potential confounding introduced by using gene expression data, we construct a model without using expression features. The HRGs and LRGs predicted by this model were compared. The results show highly consistent qualitative trends with those obtained from the full model, indicating that the observed biological patterns are not driven by expression features used during training (S7 Fig).

### The HRGs identified by MOGT compared with other methods

**Fig 4:**
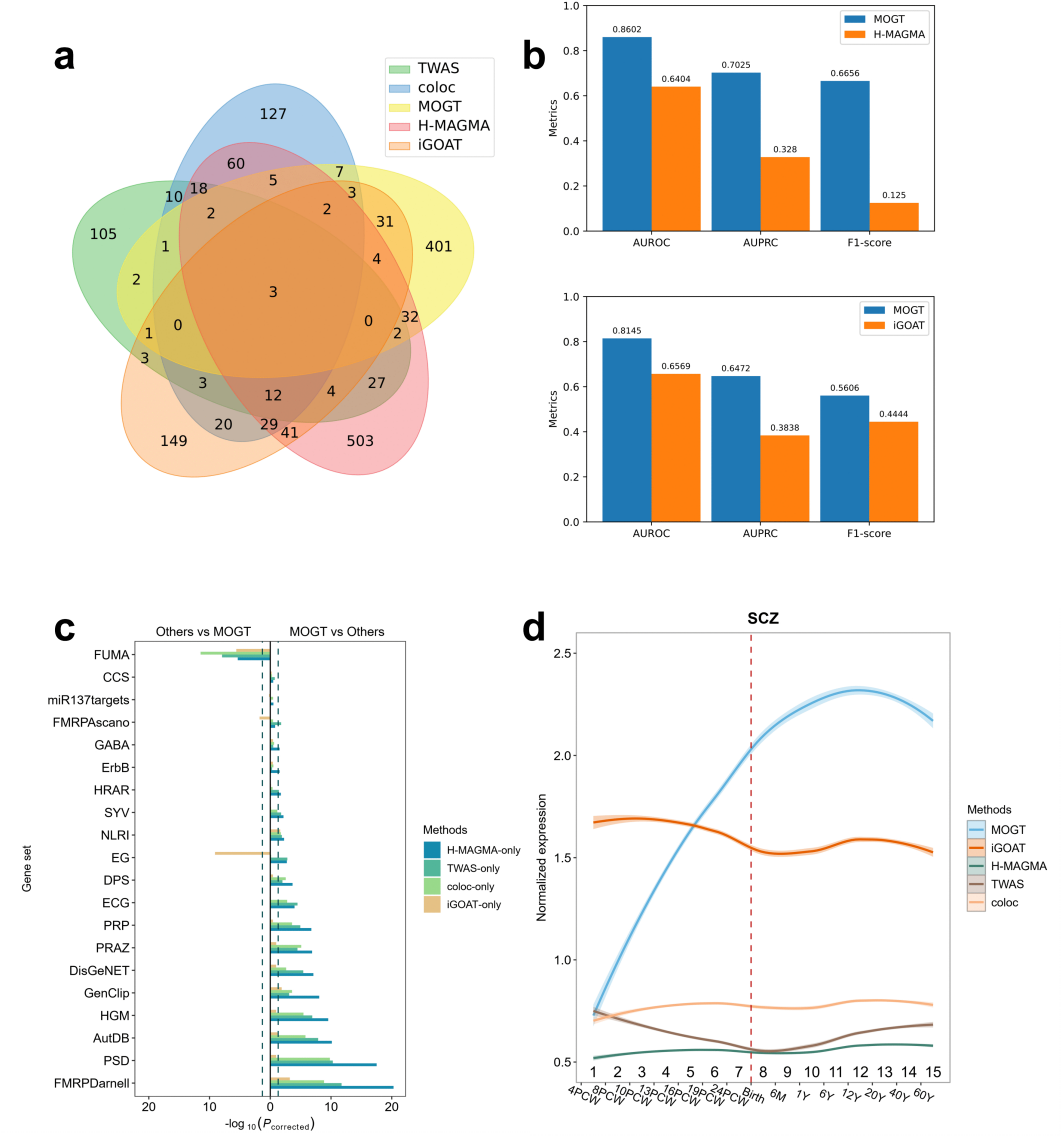
Comparing MOGT with other risk gene annotating methods, iGOAT, coloc, TWAS, and H-MAGMA. **(a)** The number of overlapping genes between SCZ-associated HRGs identified by MOGT and those identified by iGOAT, coloc, TWAS, and H-MAGMA. **(b)** Performance comparison of each model on the SCZ risk gene annotation task. **(c)** Gene set enrichment analysis of MOGT-only, iGOAT-only, TWAS-only, coloc-only, and H-MAGMA-only. The plot on the right shows the gene sets enrichment of HRGs MOGT-only compared to other methods, and the left represents the gene sets enrichment of HRGs only predicted by other methods compared to MOGT-only. The enrichment analysis was assessed by one-sided Fisher’s exact test with BH correction, and the dashed line means −log10(0.05). **(d)** Normalized expression levels of the MOGT, iGOAT, TWAS, coloc, and H-MAGMA in brain developmental stages. The dashed line represents birth. The shaded areas represented the 95% confidence intervals of the expression levels.

Taking SCZ as an example, we compared the HRGs identified by MOGT with genes predicted by other methods, including two eQTL-based approaches, coloc [28] and TWAS [29], a gene-level genome-wide association analysis, H-MAGMA [30], and an unsupervised Bayesian framework, iGOAT [5]. Both TWAS and coloc were obtained from the adult human DLPFC region. To make the results comparable, we performed H-MAGMA derived from the adult brain (v1.08) and MOGT on a core Psychiatric Genomics Consortium (PGC) dataset [31]. Finally, we obtained 496, 305, 302, 193, 744 HRGs for MOGT, iGOAT, coloc, TWAS, and H-MAGMA, respectively. We detected a significant overlap between the HRGs predicted by MOGT and four other sets of genes. Precisely, we found that the HRGs were significantly more likely to overlap with the genes predicted by four other methods than the LRGs (Fig 4a. *p*_*corrected*_ = 6.00 × 10^−3^ [coloc]; *p*_*corrected*_ = 1.55 × 10^−2^ [TWAS], *p*_*corrected*_ = 1.89 × 10^−4^ [H-MAGMA]; *p*_*corrected*_ = 2.20 × 10^−8^ [iGOAT], one-sided Fisher’s Exact test with BH correction). The performance of MOGT was compared to iGOAT (MOGT average AUROC = 0.8145, iGOAT AUROC = 0.6569) and H-MAGMA (MOGT average AUROC = 0.8602, H-MAGMA AUROC = 0.6404) in the hold-out test set (Fig 4b).

MOGT predicted 446 HRGs that are not predicted as risk genes by H-MAGMA, which were named MOGT-only genes. In reverse, 693 genes are predicted as risk genes by H-MAGMA but missed by MOGT, and these genes are named as H-MAGMA-only. Compared to the H-MAGMA-only genes, MOGT-only genes are significantly (*p*_*corrected*_ < 0.05) enriched in 16 gene sets related to SCZ. When we used MOGT-only genes as background genes, HMAGMA-only genes are significantly (*p*_*corrected*_ < 0.05) enriched in one SCZ-related gene set (Fig 4c).

MOGT was further compared to other methods. The MOGT-only genes are significantly (*p*_*corrected*_ < 0.05, one-sided Fisher’s Exact test with BH correction) enriched in 15, 11, and 3 SCZ gene sets when the TWAS-only gene set, the coloc-only gene set, and the iGOAT-only gene set are used as background genes, respectively. In comparison, when MOGT-only genes are used as background, the TWAS-only gene set, the coloc-only gene set, and the iGOAT-only gene set are significantly enriched in 1, 1, and 3 SCZ gene sets (Fig 4c), respectively. These results indicated that MOGT is more sensitive in predicting disease genes than other methods.

We also compared the expression levels of HRGs during the developmental stages of the brain with the output of the four methods (Fig 4d). We noted that the HRGs predicted by MOGT exhibited higher expression levels than HRGs identified by other methods during brain development. In summary, by comparing with the other four methods, we proved that MOGT is more powerful in predicting brain disorder risk genes.

### Applications of MOGT in drug discovery

**Fig 5:**
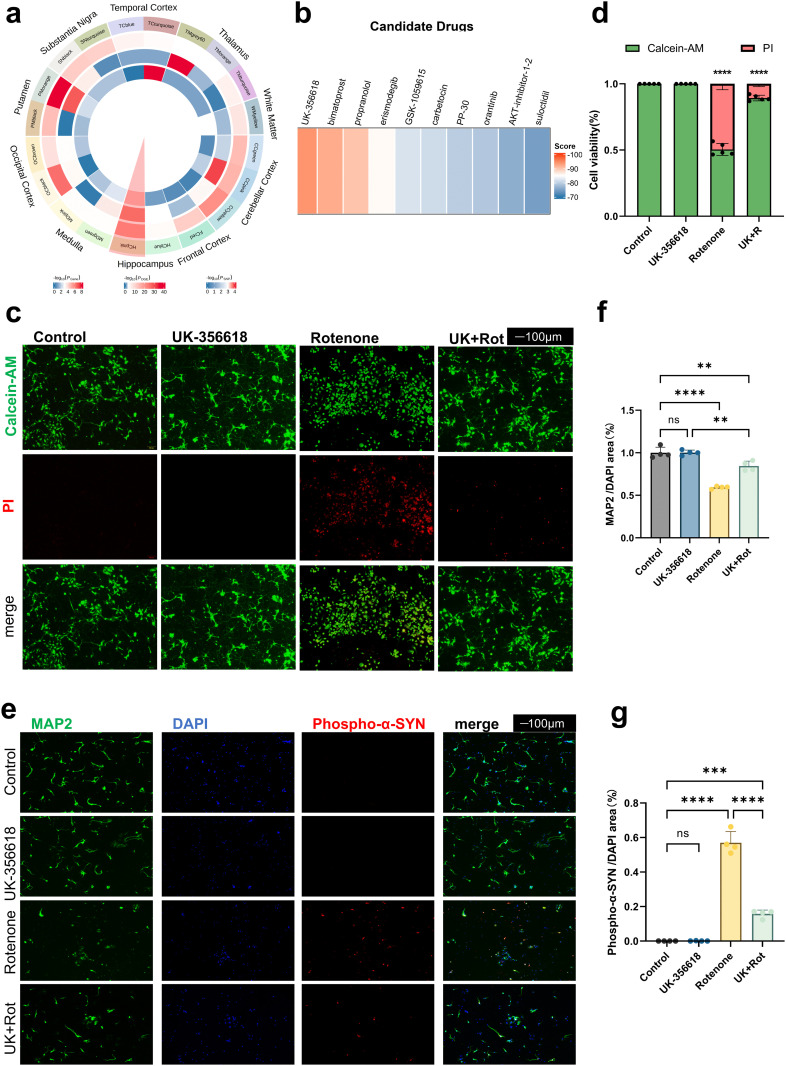
Application of MOGT in drug repurposing for Parkinson’s disease. **(a)** Specific co-expressed modules (SCMs) enriched by HRGs predicted by MOGT. From the outer ring to the inner, the circles sequentially represent the brain regions, the module names, the enrichment of HRGs, the enrichment of DGEs, and the enrichment of PD-associated SNPs. The enrichment analysis was assessed by means of one-sided Fisher’s exact test with BH correction. **(b)** Top 10 compounds with the lowest connectivity scores discovered through CMAP. **(c)**Calcein-AM/PI staining of primary murine midbrain neurons after pretreated for 2 hours with UK-356618 and then incubated with rotenone and UK-356618 for 24 hours. Green: Calcein-AM, Calcein acetoxymethyl ester; Red: PI, Propidium iodide. (scale bar = 100 μm) **(d)** After passing the normality and homogeneity of variance tests on the fluorescence intensity data shown in **(c)**, statistical analysis was performed using two-way ANOVA (n = 5; p < 0.0001). **(e)** Double immunofluorescence staining of MAP2 and phospho-α-synuclein in the primary murine midbrain neurons after treatment (scale bar = 100 μm). **(f)** Quantification of the fluorescence area ratio of MAP2/DAPI was performed as shown in **(e)**, and the fluorescence mean values were subjected to one-way ANOVA (n = 4; p < 0.0001). **(g)** Quantification of the fluorescence area ratio of phospho-α-SYN/DAPI was performed as shown in **(e)**, and the fluorescence mean values were subjected to one-way ANOVA (n = 4; p < 0.0001). MAP2: Microtubule Associated Protein 2; Phospho-α-SYN: phospho-α-synuclein (Ser129); UK + Rot: Pretreated for 2 hours with UK-356618 and then incubated with rotenone for 24 hours. P value: > 0.1234 (ns indicates no significance), < 0.0332 (*), < 0.0021 (**), < 0.0002 (***), < 0.0001 (****). Values are means ± SEM. Data are representative of at least three independent experiments.

MOGT has identified many HRGs that are not reported as disease-associated. These genes have the potential to be used for drug discovery. Application of MOGT has identified 81 PD-associated HRGs. These genes were used for drug discovery by the approach presented in our previous study [22]. We utilized the mRNA-expression data with the GEO number GSE60862 [32–34] to construct gene co-expression networks by WGCNA [24] and DiffCoEx [25]. The analysis identified 8 CCMs modules (consensus co-expressed modules) expressed in ten brain regions and 59 SCMs modules (specific co-expressed modules) specifically expressed in one of the ten brain regions but not in the other nine brain regions (S8, S9 Tables). Among them, 20 SCMs are significantly enriched with the HRGs predicted by MOGT. We further examined the enrichment of PD-associated Differentially expressed genes (DEGs) and PD-associated SNPs in these 20 SCMs (Fig 5a). Among these modules, a module named HCpink was enriched significantly with both DGEs (*p*_*corrected*_ < 0.05, one-sided Fisher’s exact test with BH correction) and PD-associated SNPs (*p*_*corrected*_ <0.05, one-sided Fisher’s exact test with BH correction). Thus, HCpink was used for drug repurposing (Supplementary Methods). Totally, 10 upregulated and 19 downregulated genes from this gene co-expression network are input into the CMAP dataset (S10 Table). Compounds are ranked according to connectivity scores, and the top 10 compounds with the lowest connectivity scores are suggested as candidates for PD treatment (Fig 5b, S11 Table). We analyzed the top ten highest-scoring compounds. Among them, propranolol [35,36] (PubChem CID: 4946) has already been investigated in the context of Parkinson’s disease, while Bimatoprost (PubChem CID: 5311027) and Erismodegib (PubChem CID: 24775005) show significant irritancy or health hazard according to the PubChem database based on GHS (Globally Harmonized System of Classification and Labelling of Chemicals). The other two compounds, UK-356618 and GSK-1059615, are not invested in PD.

To verify the therapeutic effect of UK-356618 on PD, we chose rotenone to establish a PD model in a hippocampal neuronal cell line (HT-22 cells) and mouse primary neurons (Methods, S8a Fig). UK-356618 did not exhibit toxicity across the tested concentration range (S8b Fig). We further conducted cytological experiments around the IC₅₀ of 5.9 nM for UK-356618 [37]. Compared to the rotenone-exposed PD model, the cells pre-treated with 6 nM UK-356618 for 2 hours and subsequently co-incubated with UK-356618 and rotenone for 8 hours displayed a significant increase in viability (S8c Fig).

The effects of UK-356618 on the PD primary neuronal model were further tested by leveraging the concentration-time optimum determined in HT-22 cells. The primary neuronal viability was assessed using Calcein-AM/PI staining. The result indicated that the rotenone-induced loss of neuronal viability was significantly restored through pre-treatment with UK-356618 (Fig 5c,5d). Concurrently, we observed that the synaptic loss triggered by rotenone in primary neurons was significantly ameliorated by UK-356618.

To confirm our findings, we performed staining on primary neurons to evaluate synaptic integrity and the accumulation of misfolded, aggregated α-synuclein (phospho-α-Syn) [38]. The number and morphology of neuronal dendritic branches were assessed using MAP2 (microtubule-associated protein 2). Dual-immunofluorescence staining showed that rotenone exposure elicited pronounced synaptic loss and a concomitant increase in phospho-α-synuclein (Ser129) aggregates in primary neurons. Treatment with UK-356618 partially preserved synaptic integrity and attenuated α-synuclein aggregation (Fig 5e-5g).

**Fig 6:**
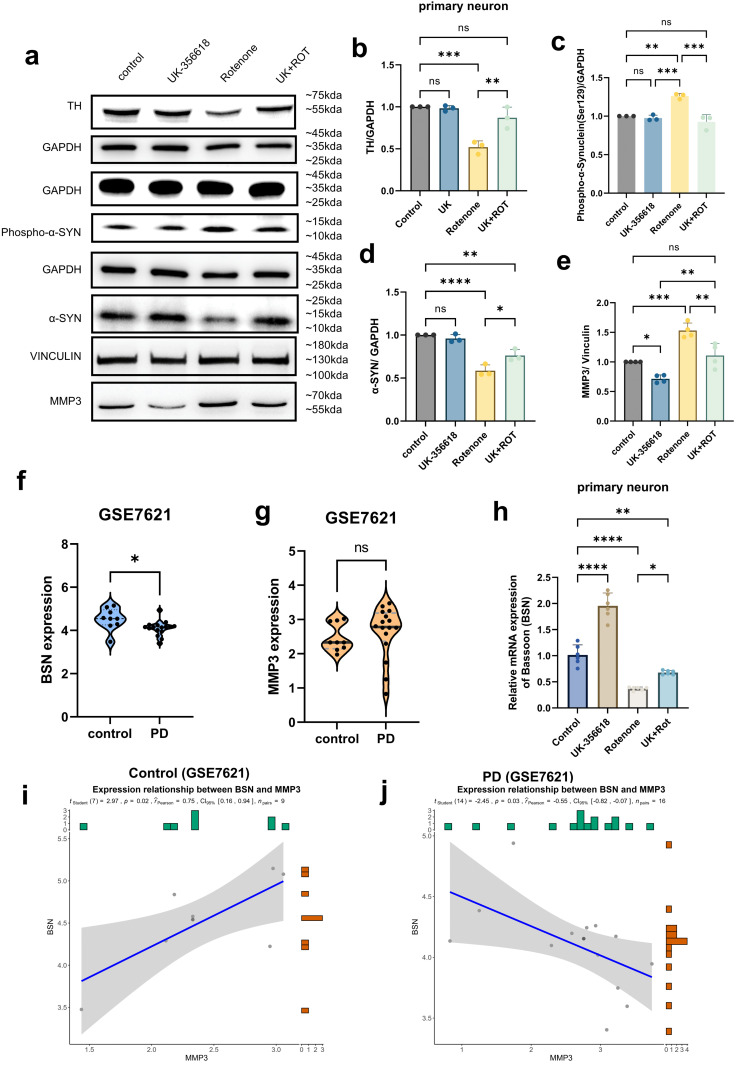
The effect of UK-356618 in restoring abnormal gene expression in PD primary neuron model. **(a)** Immunoblot analysis of lysates from primary murine midbrain neurons after pretreatment for 2 hours with UK-356618 and then incubated with rotenone and UK-356618 for 24 hours. **(b-e)** Quantification of TH, phospho-α-synuclein, α-synuclein protein, MMP3 level shown in **(a)**, statistical analysis was performed using two-way ANOVA (n = 3; p < 0.0001). **(f, g)** *BSN* and *MMP3* mRNA expression of substantia nigra from postmortem human brain of Parkinson’s disease patients (GSE7621) (9 replicates for the controls and 16 replicates for the Parkinson’s disease patients were used. Both cohorts included males and females; P_BSN_ value = 0.0199, P_MMP3_ value = 0.5564). **(h)** Bassoon(*BSN*) mRNA expression of primary neurons after pretreatment for 2 hours with UK-356618 and then incubated with rotenone and UK-356618 for 24 hours (n = 6; p < 0.0001). **(i, j)** Correlation analysis between *MMP3* and *BSM* transcript abundance. Scatter plot of normalized RNA-seq counts (log_2_ TPM) for *MMP3* versus *BSN* across n = 9 control samples and n = 16 Parkinson’s disease samples. The solid blue line denotes the linear regression fit; the shaded band represents the 95% confidence interval. P value: > 0.1234 (ns indicates no significance), < 0.0332 (*), < 0.0021 (**), < 0.0002 (***), < 0.0001 (****). Values are presented as means ± SEM. Data are representative of at least three independent experiments.

Subsequently, we conducted Western blot–based quantitative analyses in primary neuronal whole-cell lysates. The protein level of the dopaminergic neuron-specific marker tyrosine hydroxylase (TH) in rotenone-exposed cells was significantly decreased compared to the controls (P = 0.0002). This decrease was markedly reversed by UK-356618 pretreatment (P = 0.0021) (Fig 6a, 6b). We observed that rotenone treatment significantly increased the level of phospho-α-synuclein (Ser129) (P = 0.0016) while substantially decreasing the total soluble α-synuclein (P < 0.0001). These changes were effectively reversed by treatment with UK-356618, restoring α-syn levels (P = 0.0168) and lowering phospho-α-synuclein abundance (P = 0.0003) (Fig 6a, 6c, 6d). These protein-level changes were corroborated by immunofluorescence staining (Fig 5e). The trends in these protein expressions were also validated in HT-22 cells (S8d Fig).

UK-356618 (Compound 4j) is a potent and selective inhibitor of matrix metalloprotease-3 (MMP3) [37]. Here, we found that UK-356618 pretreatment effectively reduced MMP3 to near-control levels (Fig 6a, 6e, P = 0.0022). Moreover, UK-356618 restored the abnormal expression of bassoon (BSN), a protein highlighted by our analysis (Fig 5b). Together with recent genetic evidence [39], our study suggests BSN is a molecule of interest in PD. To further validate this finding, the expressions of *BSN* in control substantia nigra tissue (n = 9) and PD substantia nigra tissue (n = 16) (from the GEO dataset GSE7621) were examined (Fig 6f, 6g). *BSN* exhibited significantly lower expression in PD samples compared to the controls (P = 0.0199). In the PD group samples, the expression level of *MMP3* showed an upward trend but was not significant. *MMP3* is merely used as a target for drugs, rather than being a treatment target for PD. BSN and MMP3 mRNA transcript levels showed a significant positive correlation (r = 0.75, P = 0.02) in control midbrain tissue (Fig 6i). In contrast, in PD samples (Fig 6j), this relationship was reversed to a significant negative correlation (r = -0.55, P = 0.03). The observed changes in the MMP3–BSN interaction were associated with PD‑relevant cellular phenotypic readouts.

BSN mRNA in primary neurons was evaluated by RT-qPCR (Fig 6h). As shown in Fig 6a and 6e, the expression of BSN was downregulated after treatment with rotenone (P < 0.0001) compared to control. The rotenone-induced down-regulation was significantly reversed (P = 0.0148) by pre-treatment with UK-356618, restoring BSN expression to 66.84% of control values.

We also conducted cytological research on GSK-1059615 and provided a detailed description in the supplementary materials.

## Discussion

Discovering new brain disorder genes and elucidating the mechanisms of brain disorders are crucial for the precise treatment of brain disorders. Integrating multi-omics data of genes and their interactions can effectively represent genes. Combining multi-omics information graphs with GWAS findings can effectively identify disease risk genes.

Here, we introduced a model based on graph transformer and prototype learning, MOGT, which identified high-risk genes (HRGs) associated with various neurological conditions. MOGT differs from other methods in three main ways: (1) The biological network graph of MOGT considers SNP-SNP interactions as edge relationships rather than PPI networks. (2) MOGT introduced prototype learning to the transformer-based graph neural network because genes with similar functions might be closer in graph representation and tend to belong to the same category, enhancing the ability to capture the most significant gene patterns. We maximize the mutual information between nodes and prototypes by minimizing the contrastive loss. (3) MOGT bridges GWAS findings and the biological network of brain disorders to identify novel risk genes. Hence, MOGT can precisely locate risk genes from a massive pool of candidate genes around disease-related loci, increasing the possibility of discovering disease risk genes. Compared to the existing models, it achieved better performance in the risk gene prediction task. We found that gene expression in brain regions was the most important omics feature, greatly affecting the predictive performance of MOGT. This may be because the clinical manifestations of brain disorders (such as cognitive deficits, emotional instability, hallucinations and delusions, negative symptoms) are usually closely related to structural changes in specific brain regions or abnormal circuit function. [40–42] These neurobiological abnormalities largely explain the generation and manifestation of symptoms. With the expansion of genomic data and the discovery of GWAS loci, the exploration of disease mechanisms and the identification of risk genes will be greatly improved.

We applied MOGT to five brain disorders and identified HRGs for each brain disorder. The HRGs predicted by MOGT exhibited greater tissue specificity in the brain tissues than LRGs (Figs S3, 3a). In particular, we observed that HRGs in AD and BP were significantly enriched in the LV and atrial appendage than LRGs, and AD and SCZ were significantly enriched in the pituitary than LRGs. These results are highly consistent with previous studies. Subclinical alterations in the LV might contribute to cognitive decline by aggravating tau phosphorylation and neurodegeneration in cognitively normal individuals [43] A matched case–control study revealed that patients with BP exhibited greater LV concentric hypertrophy and cardiac dysfunction than healthy controls [44]. The hypothalamic–pituitary–adrenal axis (HPA axis) is the major stress response pathway in the body, and dysregulation of the HPA axis and cortisol levels is associated with AD and SCZ [45–48]. The midbrain is involved in the pathophysiology of various brain diseases, including SCZ [49–51], AD [52], PD [53,54], and BP [51,55]. We found that HRGs associated with SCZ, BP, AD, and PD have more pronounced cell-type specificity in neuronal cells in midbrain tissues, including GABAergic neurons, Inhibitory neurons, Excitatory neurons, Dopaminergic neurons (DaN), and CADPS2-overexpressing neuronal cells, compared with LRGs (Fig 3b). Moreover, the expression levels of HRGs during brain development stages exhibited similar expression patterns, which suggests that the five brain disorders may have a similar pathogenesis mechanism.

Taking SCZ as an example, we compared MOGT with a Bayesian Framework (iGOAT), two eQTL-based gene annotation tools (TWAS and coloc), and gene-level genome-wide association analysis(H-MAGMA). MOGT showing significantly better performance than current approaches (MOGT average AUROC = 0.8602, H-MAGMA AUROC = 0.6404; MOGT average AUROC = 0.8145, iGOAT AUROC = 0.6569), and the MOGT-only genes are significantly enriched in more SCZ gene sets compared to other methods. These results indicated MOGT is a more powerful tool for detecting brain disorder-related genes.

Our ultimate goal is to guide the development of effective treatments. To this end, the genes identified by MOGT were used to discover drug candidates, leading to the identification of 10 candidate compounds for PD. It is important to clarify that the direct drug target, MMP3, was not itself a high-risk gene (HRG) predicted by MOGT. This observation highlights the rationale of our module-based strategy. Rather than focusing on a single predicted gene, our pipeline operated at the network level: MOGT identified HRGs (e.g., BSN), which were enriched within a specific co-expression module (HCpink). This module, representing a disease-associated functional unit, was then considered as a potential target for intervention. By screening for compounds that could reverse the expression signature of the HCpink module, we identified UK-356618, a known MMP3 inhibitor. In our experimental system, treatment with UK-356618 improved cellular phenotypes and was accompanied by changes in the expression levels of both MMP3 and BSN. These observational results indicate that there may be a potential functional association between the predicted genes (BSN), drug targets (MMP3), and the observed phenotypic improvements at the module level. This strategy reflects a shift from focusing solely on individual genes to considering the broader disease-associated network context. Within the HCpink network, MMP3 may act as an influential regulatory node. Although it was not predicted as a genetic risk gene, its activity may still affect the overall state of the module. Therefore, inhibition of MMP3 by UK-356618 could potentially contribute to partial normalization of the dysregulated network. Such an approach may provide a practical way to modulate disease-associated pathways without directly targeting core risk genes that are often difficult to pharmacologically manipulate, such as BSN.

In our models, we observed that UK-356618 exerted neuroprotective effects under injury conditions. Injured neurons showed increased MMP3 expression and a negative correlation with BSN levels. Previous studies have reported that elevated MMP3 can activate inflammatory cascades [56]. Based on these observations and prior reports, one possible explanation is that inhibition of MMP3 may attenuate inflammatory signaling and thereby reduce dopaminergic neuron vulnerability [57] and neuronal loss [58]. However, these interpretations remain speculative and require further experimental validation to establish causal mechanisms. However, these findings are consistent with previous research results, which indicate that MMP3 may play a role in the process of neurodegeneration. [59,60].

Bassoon (BSN), a major presynaptic scaffold protein [61], has been implicated in multiple aspects of synaptic organization and function, including presynaptic ubiquitination [62], vesicle exocytosis [63], and homeostatic plasticity [64]. It is noteworthy that following the completion of our study, a recent genetic study reported an association between BSN gene mutations and gait and motor impairments in PD patients [39]. This independent evidence from a population genetics perspective is consistent with the involvement of BSN in PD-related processes. Our work focuses on the cellular model level, where BSN loss was associated with alterations in PD-relevant cellular phenotypes, including neuronal activity, synaptic function, and α-synuclein accumulation. Together, these findings provide complementary insights into the possible involvement of BSN in PD at the molecular and cellular levels.

Overall, our framework not only facilitates the identification of candidate neuroprotective compounds but also generates testable hypotheses regarding potential interactions among proteins within disease-relevant networks. Further functional studies will be required to clarify the precise mechanisms underlying these relationships.

Despite the above advantages, MOGT may still face potential limitations. Firstly, in this study, the strategy used for constructing negative datasets includes removing those interacting with positive genes. Our original motivation for this negative sampling strategy was to reduce label ambiguity. Genes that interact with known risk genes are more likely to be functionally related and may represent unlabeled or weakly associated risk genes rather than true negatives. Excluding such genes was intended to construct a cleaner negative set, avoid introducing false negatives during training, and empower the prediction ability of the model. However, this design choice may artificially amplify the separability of positive and negative nodes in the graph. Secondly, we used the ± 1 Mb window to define the candidate genes regulated by SNPs. Although the regulatory effects can be distal, GWAS-derived SNPs are not used as model input features, but only to define a candidate gene set that constrains the search space for high-risk gene identification. As such, the ± 1 Mb window does not determine model learning, but rather serves as a conservative filter to include genes with plausible genetic proximity to disease-associated loci. Thus, using a broader window increases sensitivity at the cost of specificity, while a narrower window risks excluding relevant genes. Nevertheless, incorporating long-range regulatory interactions represents an important future direction. Thirdly, the candidate gene space is defined by SNP thresholding and window size. MOGT was trained to improve the performance in evaluating the associations between the candidate genes and diseases. Consequently, the presence of correlated SNPs primarily affects the representation of loci within the candidate gene pool, rather than the prediction of the model. Sensitivity analysis across different window sizes demonstrated that the prioritization results are largely robust to variations in the candidate gene set. However, accounting for LD structure, lead SNPs, and ancestry heterogeneity in GWAS summary statistics can further refine candidate gene selection and improve recall in identifying disease-associated genes. Fourthly, MOGT only considers the interactions between genes, but in fact, genes are also affected by other biological molecules, such as transcription factors (TFs). We hope to incorporate other bioinformatic molecules into the bioinformatic network in future work to improve the ability to identify disease risk genes. Furthermore, data from single-cell sequencing encodes information about genes and the tissue microenvironment, which helps us understand the interactions between genes. Effective integration of single-cell sequencing data may help identify disease-causing genes. Finally, the ability of MOGT to identify HRGs is partially dependent on the power of the original GWAS. Hence, further development of GWAS studies should dramatically improve the predictive power of MOGT.

In conclusion, we have proposed a general framework, MOGT, which integrates heterogeneous genomic data with graph representation learning to allow the prediction of disease-associated genes. The application of MOGT to both psychiatric disorders and neurodegenerative/neurological diseases revealed its ability to identify disease-associated genes. Further research indicated that HRGs predicted by MOGT may aid in the discovery of drug candidates for diseases and in revealing their underlying mechanisms. MOGT is flexible in terms of input features, showing that our framework could be applicable to other diseases by leveraging disease‑specific multi‑omics data.

## Methods

### Ethics statement

This animal experiment was approved by the Sun Yat-sen Memorial Hospital, Sun Yat-sen University, with approval NO. of AP20250134. The animal experiments and experimental procedures have all been appraised in accordance with the guidelines for the Welfare and Ethics Review of Laboratory Animals, and are strictly carried out in compliance with the regulations on the Management of Laboratory Animals in Guangdong Province.

### Data source and processing

MOGT is a brain disorder prediction framework built on the attributed graph. It integrated multi-omics data and SNP–SNP interaction networks. The integrated multi-omics data included differential expression (DE), enhancer-promoter interactions (EPI) in patients and controls, and gene expression in five brain regions (Parietal Lobe, Frontal Lobe, Temporal Lobe, Cerebellum, and Occipital Lobe) in adolescents and adults. We described the details of the multi-omics data collection associated with each brain disorder, as shown in Supplementary Methods. For each fold, all data preprocessing steps (including normalization and missing data imputation) were fitted using only the training data, and the fitted parameters were subsequently applied to the validation and test sets. This design prevents any information from the validation or test sets from influencing the preprocessing procedure. For SNP–SNP interaction networks, we collected five networks for SCZ, BP, AD, PD, and MG, respectively. In addition, we also collected gene annotations as ground-truth values (1 for diseases related and 0 for non-diseases related).

**Collection of positive and negative training samples.** We firstly collected genes related to brain disorders from four databases, Human Phenotype Ontology (HPO, https://hpo.jax.org/), Online Mendelian Inheritance in Man (OMIM, https://omim.org/), Disease Gene Network (DisGeNet, https://disgenet.com/), and GeneCards(https://www.genecards.org/). Then, we excluded genes with low expression in brain regions by using Human Protein Atlas (https://www.proteinatlas.org/), and the remaining genes were used as positive samples. For genes with low expression in brain regions in Human Protein Atlas, we excluded genes that interacted with positive samples by using BioGrid (https://thebiogrid.org/), and the remaining genes were used as negative samples. The number of positive and negative samples for each brain disorder is shown in the S12 Table.

From the positive and the negative data, 80% were randomly selected as the training set for each brain disorder. The remaining genes were used as hold-out test sets.

**Defining candidate genes.** The candidate genes are those with 1Mb distance to the SNP index (*p* < 5 × 10^−8^) in disease-associated GWAS. The significant SNPs (P < 5 × 10^−8^) were directly taken from the original published GWAS for each disease and only used to select candidate genes. GWAS-derived SNPs are used for selecting disease-specific candidate genes, with the purpose of restricting the search space. Varying the SNP threshold primarily affects the size of the candidate gene pool, but not the learned model parameters or decision function of MOGT. In this study, the ± 1 Mb window was adopted as a pragmatic and widely used heuristic to associate GWAS variants with nearby genes, particularly in the absence of disease-specific, high-resolution regulatory maps. This window size is commonly used in GWAS-based gene prioritization studies to balance coverage of local regulatory effects and control of false-positive gene inclusion [4,5,65]. In total, we collected 287, 112, 397, 181, and 75 SNPs for SCZ, BP, AD, PD, and MG, respectively. The number of unique SNPs used for this study associated with each disorder is shown in S13 Table, and disease-specific lists of significant SNPs are shown in S14 Table. In general, the number of candidate risk genes for SCZ, BP, AD, PD, and MG was 1378, 466, 1112, 595, and 214, respectively.

**SNP–SNP interaction networks.** We collected the epistasis interactions between SNPs from a previous study [5], which estimated SNP-SNP interactions between genes for brain disorders under a dominant–dominant model (DDM) by the hypergeometric test. Under the DDM, three genotypes of each SNP, the majority homozygous, heterozygous, and minority homozygous, were denoted MM, Mm, and mm, respectively. A SNP is encoded as MM = 2, Mm = 1, and mm = 0. Genotype and phenotype data utilized to establish SNP-SNP interactions were downloaded from the UK Biobank (UKB), including 500,000 individuals. Based on trait-specific SNP-SNP interactions, the interaction between genes $g_{i}$ and $g_{j}$ was defined as SNP-SNP interaction negative logarithm between SNP $S_{i}$ and $S_{j}$, if$\;S_{i}$ and $S_{j}$ had the highest significant correlation level with a given disease among all SNP pairs located in 1kb of ${\text{g}}_{\text{i}}\;$and$\;{\text{ g}}_{\text{j}}$. For each brain disorder, we kept the interactions with scores at the top 1%. To validate the reliability of MOGT on other SNP-SNP interaction networks, we tested MOGT on the top1%, top2%, top5%, and top10% scores of SNP-SNP interactions.

### Prototype learning

MOGT introduces prototype learning to enhance the ability to capture the most significant gene patterns that can aid in identifying the genes within each class. The prototype layer allocated a fixed number of prototypes for each class. We use *n* to denote a node and ${\text{V}}_{c}=\left\{\overset{\text{1}}{\underset{c}{\text{v}}},\overset{\text{2}}{\underset{c}{\text{v}}},..,\overset{\text{M}}{\underset{c}{\text{v}}},\right\}$ to denote the prototypes for a given class *c*, where *M* is the number of prototypes for each class, and each prototype $\overset{m}{\underset{p_{c}}{v}}$ is a parameter vector that serves as the latent representation of the prototypical part of class *c*. For *J* classes, the total number of prototypes K=J×M. The prototype layer consists of a set of prototypes $V_{p}=\left\{\overset{m}{\underset{c}{v}}|c=1,...,J;m=1,...,M\right\}$.

Recent studies on contrastive learning have proven that minimizing contrastive loss is equivalent to maximizing the mutual information between two variables. Therefore, our goal is to maximize the similarity between a node and its positive prototypes, while minimizing the similarity with the negative prototypes. Hence, the contrastive loss is given by,


$$\begin{array}{c} L_{pl}=-\frac{\text{1}}{N}\sum_{i=\text{1}}^{N}log\frac{\sum_{\text{m}:\overset{\text{m}}{\underset{p}{v}}\in V_{y_{i}}}exp\left(\frac{g\left(\overset{i}{\underset{n}{v}},\overset{m}{\underset{p}{v}}\right)}{T}\right)}{\sum_{j:\overset{j}{\underset{p}{v}}\notin V_{y_{i}}}exp\left(\frac{g\left(\overset{i}{\underset{n}{v}},\overset{j}{\underset{p}{v}}\right)}{T}\right)}, \end{array}$$ (1)

where T is the temperature hyperparameter, N denotes the number of nodes in a batch, m indicates indices of positive samples, and j indicates indices of negative samples. $V_{y_{\text{i}}}$ is the set of prototypes that belong to the class $y_{i}$. The similarity score between the prototype $v_{p}$ and the embedding $v_{n}$ is as follows:


$$\begin{array}{c} g\left(v_{n},v_{p}\right)=log\left(\frac{\sum {\left(v_{n}-v_{p}\right)}^{\text{2}}+\text{1}}{\sum {\left(v_{n}-v_{p}\right)}^{\text{2}}+\varepsilon }\right), \end{array}$$ (2)

### Transformer-based gene prediction model for brain disorders

MOGT is built on a transformer-based graph neural network. We applied the layer normalization (LN) before the second Layer Graph Transformer and the max-pooling. The two fully connected linear layers are used to make the final prediction. The dropout layer is used to prevent overfitting. We used focal loss for disease risk gene prediction. which was defined as follows,


$$\begin{array}{c} FL\left(p_{t}\right)=-\alpha_{t}{\left(\text{1}-p_{t}\right)}^{\gamma }log\left(p_{t}\right), \end{array}$$ (3)

whereα (α∈[0,1]) denoted the weighting factor, γ(γ≥0) denoted the tunable focusing parameter, and ${\text{p}}_{\text{t}}$ was defined as,


$$\begin{array}{c} p_{\text{t}}=\left\{ \begin{array}{c} p,y=\text{1}\\ \text{1}-p,otherwise \end{array}\right.\;\;\;, \end{array}$$ (4)

where y denoted the ground-truth class, p denoted the model’s estimated probability for the class with label y=1. In the experiments, α was set to 0.25 and γ was set to 2.

Finally, we define the objective of our model as the sum of the losses as follows:


$$\begin{array}{c} L_{total}=\;FL\left(p_{t}\right)+L_{pl}, \end{array}$$ (5)

### Training the model

In the training process, we initially divided the labelled data into training and testing sets by stratified sampling for all datasets and applied 5-fold cross-validation (CV) to validate the model. To avoid overfitting and over-smoothing, we implemented an early stop strategy that would terminate training if the validation performance did not improve for 50 consecutive epochs. To improve model robustness and reduce overfitting, the dropout rate was set to 0.4 for the modules. The prototype layer allocated seven prototypes to each class. To address class imbalance, we used the Focal loss for gene classification. Finally, to keep high efficiency and stable performance, we extracted subgraphs from the input graphs. These subgraphs contained all first- and second-order neighbors of each node and served as the basis for training. We performed mini-batch neighbor-sampled training with masked supervision in which the loss is computed exclusively on nodes in the training mask, and although the neighbor sampler operates on the full graph, validation and test nodes are included only as unlabeled context without contributing labels or gradients, preventing information leakage. The threshold for identifying high-risk genes was calculated using Youden’s index in the best model in the validation set. The performance of the model in five-fold cross-validation was evaluated on a hold-out test set. The performance was evaluated by AUROC, F1-score, and AUPRC.

### Comparing MOGT with other methods

To evaluate the performance of MOGT on identifying high-risk genes in brain disorders, we compared MOGT with four other methods, EMOGI [13], HGDC [66], logistic regression, and LabelSpreading. For these two methods, EMOGI and HGDC, we maintained the same model architectures and hyperparameter settings as described in their original papers and used the same network and gene feature matrix as inputs. Feature-only baseline, implemented using logistic regression on the same multi-omics feature matrix. Graph-only baseline, implemented using LabelSpreading on the SNP–SNP interaction graph. Same model without prototype loss, constituting a true architectural ablation of MOGT. The performance of the models in five-fold cross-validation was evaluated on a hold-out test set and evaluated by AUROC, AUPRC, and F1-score.

Using SCZ as an example, we also compared the output of MOGT with the output of four high-risk gene annotation methods for brain disorders, including the Bayesian framework iGOAT, two eQTL-based approaches, coloc and TWAS, and a developed gene-level genome-wide association analysis, H-MAGMA. Both TWAS and coloc were obtained from the adult human DLPFC tissue. H-MAGMA was performed using the epigenetic data derived from the adult brain (v1.08). The input GWAS for H-MAGMA and MOGT are from a core Psychiatric Genomics Consortium (PGC) dataset of 90 cohorts of European (EUR) and East Asian (ASN) ancestry from the PGC, involving a total of 67,390 cases and 94,015 controls. This GWAS identified 300 independent SNPs (linkage disequilibrium (LD) r^2^ < 0.1) that exceeded genome-wide significance (P < 5 × 10^−8^). For MOGT, we classify genes of the training data with test scores.

### Cell-type specificity analysis

We collected single-cell transcriptomic data from two GEO datasets, GSE67835 [67] and GSE157783 [54,68,69]. GSE67835 includes 466 cells of the adult and fetal human brain at a whole transcriptome level. GSE157783 contains single-cell transcriptomics of the postmortem midbrain of six controls and five idiopathic Parkinson’s disease cases.

We evaluated the cell type specificity for HRGs by using LRGs as background, as a strategy from a previous study [27]. We assigned the mean expression level of gene *g* in cell type *c* as the expression value of gene *g* in cell type *c*. We calculated the expression proportion of gene *g* and cell type *c* as the cell-specific score. The specificity metric for gene *g* and cell type *c* is given by,


$$\begin{array}{c} S_{g,c}\;=\;\frac{\sum_{i=\text{1}}^{N_{c}}r_{g,i,c}N_{c}}{\sum_{j=\text{1}}^{w}\left(\sum_{i=\text{1}}^{N_{j}}r_{g,i,j}N_{j}\right)}, \end{array}$$ (6)

where $r_{g,i,c}$ means the expression of gene g in the *i-th* cell of cell type c; $N_{c}$ is the number of cell type *c*; and *w* is the total number of cell types. We then employed the one-sided Wilcoxon rank-sum test with BH correction to evaluate whether the HRGs predicted by MOGT carry lower P values compared to the LRGs.

### Tissue-type specificity analysis

The tissue-specificity investigation was performed using two gene expression datasets from two sources, as a strategy from a previous study [26]. Briefly, we downloaded the median expression levels, Reads Per Kilobase of transcript, per Million mapped reads (RPKM) of genes in brain tissues from GTEx (V8, https://gtexportal.org/home/datasets), and the expression levels of genes in ten brain regions from BrainEAC (http://www.braineac.org/, cerebellar cortex [CRBL], frontal cortex [FCTX], hippocampus [HIPP], medulla [MEDU], occipital cortex [OCTX], putamen [PUTM], substantia nigra [SNIG], thalamus [THAL], temporal cortex [TCTX], and intralobular white matter [WHMT]). We normalized the expression of each gene in all tissues to a density vector by dividing the total expression counts. Formally,


$$\begin{array}{c} E=\frac{V}{\sum_{i=\text{1}}^{n}v_{i}}, \end{array}$$ (7)

where $\text{V }=\;\left({\text{v}}_{\text{1}},{\text{v}}_{\text{2}},..,{\text{v}}_{\text{n}}\right)$ is the expression vector of a gene across all tissues and E is the new normalized density vector. For two discrete probability distributions ${\text{p}}^{\text{1}}$, ${\text{p}}^{\text{2}}$, the JS divergence is given by:


$$\begin{array}{c} JS\left(p^{\text{1}},p^{\text{2}}\right)=H\left(\frac{p^{\text{1}}+p^{\text{2}}}{\text{2}}\right)-\frac{H\left(p^{\text{1}}\right)+H\left(p^{\text{2}}\right)}{\text{2}} \end{array}$$ (8)

where *H* is the entropy of a discrete probability distribution $\text{H}(\text{p})\;=\;-\sum \overset{\text{n}}{\underset{\text{i}=\text{1}}{\mathrm{\backslash nolimits}}}{\text{p}}_{\text{i}}\;\text{log}\;{\text{p}}_{\text{i}}$ and $p_{i}$ is the proportion of event *i* happening. The distance between two tissue expression patterns $\;E^{\text{1}}$ and $E^{\text{2}}$, was defined as the square root of the JS divergence, $\sqrt{JS\left(E^{\text{1}},E^{\text{2}}\right)}$. The tissue specificity score of the gene with expression *V* across n tissues with respect to tissue t is given by:


$$\begin{array}{c} TS\left(E|t\right)\;=\;\text{1}-\sqrt{JS\left(E,E^{t}\right)}, \end{array}$$ (9)

where $E^{t}\;=\;\left(\overset{t}{\underset{\text{1}}{E}},\overset{t}{\underset{\text{2}}{E}},..,\overset{t}{\underset{n}{E}}\right)$ be the expression of a gene that is only expressed in tissue t, t = 1, 2, …, n and $\begin{array}{c} \overset{\text{t}}{\underset{\text{i}}{\text{E}}}=\left\{ \begin{array}{c} \text{0},\text{i}=\;\;/\;\text{t}\\ \text{1},\text{ i}=\text{t} \end{array}\right.\;\;. \end{array}$

We then employed the one-sided Wilcoxon rank-sum test to evaluate whether the HRGs predicted by MOGT carry lower P values compared to the LRGs.

### Gene set enrichment analysis

A total of 20 functional gene sets associated with brain disorders were systematically curated for this study by following the procedures described in a previous study [5]. We collected three SCZ-related gene sets(FUMA: Risk genes of SCZ predicted by FUMA; GABA: GABA_A_ receptor complex; miR.137.targets: Targets of miR-137), seven synaptic pathway-specific gene sets (CCS: Calcium Channel Synapse; FMRP.Ascano: FMRP targets; FMRP.Darnell: FMRP targets; PRAZ: Presynaptic Active Zone; PRP: Postsynaptic Proteome; PSD: Postsynaptic Density; SYV: Synaptic Vesicle) which were involved in biological processes considered to be associated with the five brain disorders, including synaptic transmission, presynaptic function, and voltage-gated calcium channel activity. We also collected SCZ-associated genes, additionally extracted from two independent biomedical databases: GenCLiP and DisGeNET, and three SCZ-associated pathways from KEGG. These curated gene sets collectively represent molecular pathways implicated in SCZ, as detailed in S15 Table (including gene set nomenclature, functional annotations, and cardinality). The gene set enrichment analysis was performed by Fisher’s Exact test. The results with *p*_*BH*_ < 0.05 were considered significant.

### Trajectories of genes associated with diseases

The spatiotemporal transcriptome data were downloaded from two sources. The first dataset from GEO with accession number GSE25219 [70–72] is composed of 1,340 brain tissue samples encompassing the entire human lifespan (age range 5 PCW–82 years) and covering 16 brain regions. The second dataset analyzed >700,000 single-nucleus RNA sequencing profiles from 106 donors during prenatal and postnatal developmental stages. [73] We classified the age of cells in the second dataset according to the developmental stage of the first dataset, which is divided into 12 developmental stages in total. A detailed description of the developmental stages for the second dataset is provided in S16 Table. For the first dataset, analysis was done on the core and unique probe sets, representing 17,565 mainly protein-coding genes, into gene-level information. The log-transformed expression values of all genes were centered around the mean expression level per sample using the function scale (data, center = T, scale = F) in R.

### Drug repurposing

The drug repurposing was performed as described in our previous study [22]. Briefly, it comprises by three main steps: (1) using gene expression data of 1,231 healthy human brain samples across 10 brain regions to construct consensus weighted gene co-expression network and differential co-expression network associated with normal brain functions; (2) identifying the co-expression modules enriched with disease-associated genes, SNPs and genes expressed significantly different in patients and controls; (3) identifying DEGs in the significant modules and aligning to the gene expression profiles perturbed by small molecular compounds in CMAP database. The GSE60862 [32–34] dataset was used to construct weighted gene co-expression networks by two different approaches, WGCNA [24] and DiffCoEx [25]. The WGCNA was applied to detect co-expression modules common to all ten brain regions (consensus co-expressed modules, CCMs). The DiffCoEx was used to identify gene modules specifically expressed in each of the ten brain regions compared to the other nine brain regions (specific co-expressed modules, SCMs).

The CCMs and SCMs that are enriched with the HRGs predicted by MOGT were used for further analysis. These modules were further analyzed to test their enrichment of known PD-associated SNPs and DGEs. The enrichment analysis was performed for DGEs in the GEO dataset, GSE8397 [74,75], which is RNA-seq data of the substantia nigra split into medial and lateral portions, and frontal cortex from 24 PD patients and 15 controls. We collected PD-associated SNPs from a previous study [76], which performed the largest meta-GWAS of PD to date, involving the analysis of 7.8M SNPs in 37.7K cases, 18.6K UK Biobank proxy-cases, which have a first-degree relative with PD, and 1.4M controls. The PD-related modules were delivered to the Connectivity Map (CMAP, https://clue.io/about) to find potential PD drug candidates. The drug candidates were ranked by their connectivity scores. In this study, the top 10 compounds with the lowest connectivity scores were selected for further analysis.

### Cell culture

**HT-22 cells:** HT-22 cells (CL-0697, Pricella) were cultured in Dulbecco’s Modified Eagle Medium (DMEM) supplemented with 10% fetal bovine serum (FBS; FBSST-01033-500, OriCell) and 1% penicillin-streptomycin (15140-122, Gibco) at 37 °C in a 5% CO_2_ incubator.

**Primary midbrain neurons:** Primary neurons were isolated from the ventral mesencephalon of postnatal day 0–3 C57BL/6J mice (Guangdong Medical Laboratory Animal Center). Brain tissues were dissected on ice in D-Hanks solution (PB180321, Pricella), digested with 0.25% trypsin (25200072, Gibco), and triturated to obtain a single-cell suspension. Cells were seeded at a density of 2 × 10⁶ cells per well onto poly-L-lysine (PB180523, Pricella)-coated 6-well plates and maintained in neuronal culture medium consisting of Neurobasal-A Medium (10888022, Gibco), 4% B-27 supplement (17504044, Gibco), 0.5% L-glutamine (25030081, Gibco), and 1% penicillin-streptomycin. The medium was replaced after the first 6 h and subsequently every 2 days, with neurons used for experiments on day 7 in vitro. All the procedures were carried out in accordance with the guidelines.All animal procedures were approved by the Animal Welfare and Ethics Committee of Sun Yat sen Memorial Hospital, Sun Yat sen University (Approval No. AP20250134) and conducted in accordance with institutional and national guidelines.

### Parkinson’s disease cell models and drug treatments

**Dose determination for rotenone:** To establish the rotenone-induced PD model, HT-22 cells were seeded in 96-well plates (5,000 cells/well) overnight and treated with a gradient of rotenone concentrations (0.25–16 μM; HY-B1756, MedChemExpress) or vehicle (0.1% DMSO) for 8 h. Cell viability was assessed by CCK-8 assay, and the concentration that reduced viability to 50% of the control (IC₅₀) was determined to be 4 μM (S7a Fig). This concentration was used for all subsequent rotenone treatments in HT-22 cells.

**Drug treatment protocols: HT-22 model:** Cells were pretreated with UK-356618 (HY-107394, MedChemExpress; 6, 12, or 24 nM) for 2 h, followed by co-treatment with UK-356618 and 4 μM rotenone for 8 h. **Primary neuron model**: Neurons were pretreated with 6 nM UK-356618 for 2 h, then co-treated with 6 nM UK-356618 and 400 nM rotenone (a concentration previously established by our group) for 8 h to assess neuroprotection.

### Cell viability assay (CCK-8)

Following drug treatments, 10 μL of CCK-8 reagent (K1018, APExBIO) was added to each well of the 96-well plate containing 90 μL of culture medium. Plates were incubated at 37 °C for 2 h, and the absorbance at 450 nm was measured using a BioTek SYNERGY H1 microplate reader (Agilent). Viability was expressed as a percentage relative to the untreated control group.

### Calcein-AM/Propidium Iodide (PI) staining

After adding a circle microscope cover glass (14mm, 801010, Nest) to the 24-well plate, we coated it overnight with PLL and directly inoculated neurons (2 midbrain/24 wells) into the pre-treated 24-well plate. Neurons are implanted into a 24-well plate at the beginning of 7 days of incubation. After undergoing the same medication treatment steps as described above, discard the medium and wash it twice with PBS. Then, add Calcein-AM (CA1630, Solarbio) dissolved in the medium for 25 minutes (37 °C, 5% CO2). Afterwards, use PBS wash twice and add PI dissolved in medium for 5 minutes. After washing with PBS twice, the fluorescence was detected via EVIDENT BX63 Upright Microscopes.

### Western blot

Using cell lysis buffer for Western and IP (P0013, Beyotime) with phosphatase inhibitor cocktail (CW2383S, CWbio) and protease inhibitor cocktail (CW2200S, CWbio) added to lyse cells. Equivalent amounts of boiled protein samples were separated by SDS–PAGE (FuturePAGE 4–20%, ET10420LGel, ACE) and transferred to PVDF membranes (ISEQ00010, Merck Millipore). After blocking with 5% BSA (BSA Albumin Fraction V, 4240GR100, BioFroxx), the membranes were incubated overnight at 4 °C with the indicated primary antibodies (Vinculin: 26520–1-AP, proteintech; GADPH: 60004–1-Ig, proteintech; TH: 25859–1-AP, proteintech; Alpha Synuclein: 10842–1-AP, proteintech; Phospho-α-Synuclein (Ser129): 23706S, Cell Signaling Technology; MMP3: ab52915, abcam) and then with horseradish peroxidase (HRP)-linked secondary antibodies (7074S, 7076S, Cell Signaling Technology) for 2 hours at room temperature. The proteins were detected using an ECL kit HRP (FD8020, Fdbio science) and visualized with a BLT GelView 6000Pro Imaging System

### Immunofluorescence staining

To stain neurons, circle microscope cover glasses with cells are rinsed with PBS and fixed with 4% paraformaldehyde. Then incubate with 1% Triton X-100 in PBS for 12 minutes before staining. Cells are rinsed and incubated with the following primary antibodies overnight at 4°C (MAP2: 1:200, ab300645, abcam; Phospho-α-Synuclein (Ser129): 1:200, 23706S, Cell Signaling Technology)

Sections were incubated with DAPI and the corresponding secondary antibodies (all from Abcam and all diluted 1:400) to visualize the primary antibodies: Dylight 488, Goat Anti-Rabbit IgG (A23220); Dylight 649, Goat Anti-Mouse IgG (A23610). Sections were mounted and imaged using an Olympus IX73 and Olympus BX63 microscope.

### RNA isolation and RT-PCR

Total RNA was isolated from primary mouse midbrain neurons with EZ-press RNA Purification Kit (B0004D, EZbioscience), according to the manufacturer’s instructions. The concentration was determined with a NanoDrop one spectrophotometer (Thermo Fisher, 840–317500), and 300 ng of total RNA was reverse transcribed using the HiScript II Q RT SuperMix for qPCR (gDNA wiper) (Vazyme, R223). The amount of cDNA was evaluated by ChamQ Universal SYBR qPCR Master Mix (Vazyme, Q711) on a Fast Real-Time PCR System (LightCycler 96, Roche) using primer sets for *BSN* (forward: 5′-CGAGGAGGAGAAGGAGATTGAC-3′; reverse: 5′-TGAGGCAGGGTAGTCCACATAC-3′) and GAPDH (forward: 5′-AGGTCGGTGTGAACGGATTTG−3′; reverse: 5′-TGTAGACCATGTAGTTGAGGTCA-3′). Differences in mRNA expression were calculated through the formula N = (2)^-ΔΔCT [77].

### Expression correlation analysis

We downloaded bulk transcriptomic data from the GEO datasets with accession number GSE7621 [78]. Then, log-transformed expression values of all genes were centered around the mean expression level per sample using the function scale (data, center = T, scale = F) in R. Outliers were defined as samples with values exceeding 1.5 times the interquartile range or both gene expression values exceeding one standard deviation. All outliers in these data were filled using the median expression value. The expression correlation analysis was performed by the function ggscatterstats [79].

### Statistical analysis

Western blots were analyzed as previously described. Relative optical density values for bands corresponding to TH, Phospho-α-SYN, α-SYN, and MMP3 were normalized to the value for GADPH or Vinculin. For cell viability assays, values for the control group were taken as 1. Statistical analyses were carried out using Prism 9.5 software (GraphPad Inc., La Jolla, CA, USA). Results were presented as means ± SD from at least three independent experiments. Statistical analyses were performed using analysis of variance, followed by a Bonferroni post-hoc correction for multiple comparisons. *P*_*value*_: > 0.1234 (ns indicates no significance), < 0.0332 (*), < 0.0021 (**), < 0.0002 (***), < 0.0001 (****).

## Supporting information

S1 Text Supplementary material to this article. (DOCX)

S1 Fig AUROC of MOGT with different numbers of SNP-SNP interactions. (TIF)

S2 Fig Ablation test AUROC performance of multi-omics data for five brain diseases. (TIF)

S3 Fig Tissue-specificity analysis on HRGs compared to LRGs in tissues from GTEx. (TIF)

S4 Fig Cell specificity analysis of HRGs compared to LRGs (Methods). “▴” denotes significant result (Fisher’s exact test *p*_*corrected*_ < 0.05).
(TIF)

S5 Fig Developmental expression trajectories of the HRGs during the entire brain developmental stages. (TIF)

S6 Fig Developmental expression trajectories of the LRGs during the entire brain developmental stages. (TIF)

S7 Fig Analysis of the expression of HRGs predicted by the model without using expression-features. (a)The overlap between the HRGs predicted by MOGT and the HRGs predicted by the model without using expression-features. (b) Tissue specificity analysis of HRGs compared to LRGs using expression profiles from BrainEAC (Methods). (c) Cell specificity analysis of HRGs compared to LRGs (Methods). “▴” denotes significant result (*p*_*corrected*_ < 0.05, Wilcoxon test, with BH(Benjamini-Hochberg) correction. (d, e) Developmental expression trajectories of the HRGs and LRGs during the entire brain developmental stages. The dashed line represents birth. The shaded areas represent the 95% confidence interval of expression levels.
(TIF)

S8 Fig The influence of UK-356618 in HT-22 and GSK-1059615 in primary astrocytes. (a) Cell viability of HT-22 detected by CCK-8 after incubating Rotenone for 8 hours (n = 6; *p* < 0.0001). (b) Cell viability of HT-22 detected by CCK-8 after incubating UK-356618 for 24 hours (n = 6; *p* = 0.3043). (c) Cell viability of HT-22 detected by CCK-8 after pretreated for 2 hours with UK-356618 6 nM and then incubated with rotenone 4μM and UK-356618 6 nM for 8 hours. (n = 6; *p* < 0.0001). (d) Immunoblot analysis of lysates from HT-22 after pretreatment for 2 hours with UK-356618 and then incubated with rotenone and UK-356618 for 8 hours. (e, f) Cell viability of primary astrocytes detected by CCK-8 after incubating Rotenone for 24 hours and 48 hours (n = 6; *p* < 0.0001). (g) Cell viability of primary astrocyte detected by CCK-8 after incubating GSK-1059615 for 24 hours (n = 6; *p* < 0.0001). (h) Cell viability of primary astrocytes was detected by CCK-8 after pretreatment for 24 hours with GSK-1059615 and then incubated with rotenone and GSK-1059615 for 48 hours. (n = 6; *p* < 0.0001). (i) Calcein-AM/PI staining of primary murine astrocytes after pretreatment for 2 hours with GSK-1059615 and then incubated with rotenone and GSK-1059615 for 48 hours. Green: Calcein-AM, Calcein acetoxymethyl ester; Red: PI, Propidium iodide. (scale bar = 100 μm) (j) After passing the normality and homogeneity of variance tests on the fluorescence intensity data shown in (i), statistical analysis was performed using two-way ANOVA (n = 4; *p* < 0.0001). *P*_value_: > 0.1234 (ns indicates no significance), < 0.0332 (*), < 0.0021 (**), < 0.0002 (***), < 0.0001 (****). Values are presented as means ± SEM. Data are representative of at least three independent experiments.
(TIF)

S1 Table AUROC, AUPRC, and F1-score values of the best MOGT model on the validation set on the hold-out test dataset. (XLSX)

S2 Table Methods comparison on SCZ, AD, PD, BP, and MG. Performance was evaluated on the hold-out test set using AUROC, F1-score, and AUPRC, with model selection conducted via 5-fold cross-validation.
(XLSX)

S3 Table The positive prevalence in the test set for each disorder. (XLSX)

S4 Table Ablation study on SCZ, AD, PD, BP, and MG. Performance was evaluated on the hold-out test set using AUROC and F1-score, with model selection conducted via 5-fold cross-validation.
(XLSX)

S5 Table The overlap between HRGs and LRGs predicted by MOGT on ±500kb and ±1Mb window size. (XLSX)

S6 Table The performance of MOGT on an alternative negative sample set excluding interactions from the BioGRID database. Performance was evaluated on the hold-out test set using AUROC, F1-score, and AUPRC, with model selection conducted via 5-fold cross-validation.
(XLSX)

S7 Table Number of HRGs and LRGs predicted by MOGT. (XLSX)

S8 Table Enrichment of PD-associated genes, DGEs, and SNPs in consensus co-expressed modules (CCMs) for ten brain regions. (XLSX)

S9 Table Enrichment of PD-associated genes, DGEs, and SNPs in specific co-expressed modules (SCMs) for ten brain regions. (XLSX)

S10 Table DGEs submitted to CMAP for drug candidate discovery. (XLSX)

S11 Table Drug candidates. (XLSX)

S12 Table The number of training set genes and candidate genes. (XLSX)

S13 Table SNPs associated with brain disorders are utilized in this study. (XLSX)

S14 Table The list of significant SNPs for each disease. (XLSX)

S15 Table Gene sets are associated with neurological disorders. (XLSX)

S16 Table Periods of human development and adulthood defined by single-nucleus RNA sequencing data. (XLSX)

S17 Table RNA-seq data were utilized for generating genes differentially expressed in patients and controls. (XLSX)

S18 Table Information on the 10 individuals utilized to generate enhancer-protein interactions (EPI). (XLSX)

S1 Raw Image The original Western blot data of Fig_6a (TH, GAPDH, Phospho-α-SYN). (TIF)

S2 Raw Image The original Western blot data of Fig_6a (GAPDH, α-SYN, MMP3, Vinculin). (TIF)

S3 Raw Image The original western blot data of S8d_Fig (TH, α-SYN, Phospho-α-SYN, Vinculin). (TIF)

## References

[1] Visscher PM, Wray NR, Zhang Q, Sklar P, McCarthy MI, Brown MA, et al. 10 Years of GWAS discovery: biology, function, and translation. Am J Hum Genet. 2017;101(1):5–22. 28686856 10.1016/j.ajhg.2017.06.005 PMC5501872

[2] Fan C, Chen K, Zhou J, Wong P-P, He D, Huang Y, et al. Systematic analysis to identify transcriptome-wide dysregulation of Alzheimer’s disease in genes and isoforms. Hum Genet. 2021;140(4):609–23. doi: 10.1007/s00439-020-02230-7 33140241

[3] Zhang F, Chen W, Zhu Z, Zhang Q, Nabais MF, Qi T, et al. OSCA: a tool for omic-data-based complex trait analysis. Genome Biol. 2019;20(1):107. doi: 10.1186/s13059-019-1718-z 31138268 PMC6537380

[4] Wang Q, Chen R, Cheng F, Wei Q, Ji Y, Yang H, et al. A Bayesian framework that integrates multi-omics data and gene networks predicts risk genes from schizophrenia GWAS data. Nat Neurosci. 2019;22(5):691–9. doi: 10.1038/s41593-019-0382-7 30988527 PMC6646046

[5] He D, Li L, Zhang H, Liu F, Li S, Xiu X, et al. Accurate identification of genes associated with brain disorders by integrating heterogeneous genomic data into a Bayesian framework. EBioMedicine. 2024;107:105286. doi: 10.1016/j.ebiom.2024.105286 39168091 PMC11382033

[6] Gibson G. Rare and common variants: twenty arguments. Nat Rev Genet. 2012;13(2):135–45. doi: 10.1038/nrg3118 22251874 PMC4408201

[7] Lippert C, Listgarten J, Davidson RI, Baxter S, Poon H, Kadie CM, et al. An exhaustive epistatic SNP association analysis on expanded Wellcome Trust data. Sci Rep. 2013;3:1099. doi: 10.1038/srep01099 23346356 PMC3551227

[8] Hoffmann M, Poschenrieder JM, Incudini M, Baier S, Fritz A, Maier A, et al. Network medicine-based epistasis detection in complex diseases: ready for quantum computing. Nucleic Acids Res. 2024;52(17):10144–60. doi: 10.1093/nar/gkae697 39175109 PMC11417373

[9] Cowman T, Koyutürk M. Prioritizing tests of epistasis through hierarchical representation of genomic redundancies. Nucleic Acids Res. 2017;45(14):e131. doi: 10.1093/nar/gkx505 28605458 PMC5737499

[10] Jing P-J, Shen H-B. MACOED: a multi-objective ant colony optimization algorithm for SNP epistasis detection in genome-wide association studies. Bioinformatics. 2015;31(5):634–41. doi: 10.1093/bioinformatics/btu702 25338719

[11] Duroux D, Climente-González H, Azencott C-A, Van Steen K. Interpretable network-guided epistasis detection. Gigascience. 2022;11:giab093. doi: 10.1093/gigascience/giab093 35134928 PMC8848319

[12] Kim S, Park C, Seo S. Interpretable Prototype-based Graph Information Bottleneck. In: Advances in Neural Information Processing Systems 36, 2023. 76737–48. doi: 10.52202/075280-3353

[13] Schulte-Sasse R, Budach S, Hnisz D, Marsico A. Integration of multiomics data with graph convolutional networks to identify new cancer genes and their associated molecular mechanisms. Nat Mach Intell. 2021;3(6):513–26. doi: 10.1038/s42256-021-00325-y

[14] Li H, Han Z, Sun Y, Wang F, Hu P, Gao Y, et al. CGMega: explainable graph neural network framework with attention mechanisms for cancer gene module dissection. Nat Commun. 2024;15(1):5997. doi: 10.1038/s41467-024-50426-6 39013885 PMC11252405

[15] Su X, Hu P, Li D, Zhao B, Niu Z, Herget T, et al. Interpretable identification of cancer genes across biological networks via transformer-powered graph representation learning. Nat Biomed Eng. 2025;9(3):371–89. doi: 10.1038/s41551-024-01312-5 39789329

[16] Lam M, Chen C-Y, Li Z, Martin AR, Bryois J, Ma X, et al. Comparative genetic architectures of schizophrenia in East Asian and European populations. Nat Genet. 2019;51(12):1670–8. doi: 10.1038/s41588-019-0512-x 31740837 PMC6885121

[17] Pardiñas AF, Holmans P, Pocklington AJ, Escott-Price V, Ripke S, Carrera N, et al. Common schizophrenia alleles are enriched in mutation-intolerant genes and in regions under strong background selection. Nat Genet. 2018;50(3):381–9. doi: 10.1038/s41588-018-0059-2 29483656 PMC5918692

[18] Bellenguez C, Küçükali F, Jansen IE, Kleineidam L, Moreno-Grau S, Amin N, et al. New insights into the genetic etiology of Alzheimer’s disease and related dementias. Nat Genet. 2022;54(4):412–36. doi: 10.1038/s41588-022-01024-z 35379992 PMC9005347

[19] Kim JJ, Vitale D, Otani DV, Lian MM, Heilbron K, 23andMe Research Team, et al. Multi-ancestry genome-wide association meta-analysis of Parkinson’s disease. Nat Genet. 2024;56(1):27–36. doi: 10.1038/s41588-023-01584-8 38155330 PMC10786718

[20] Reay WR, Cairns MJ. Advancing the use of genome-wide association studies for drug repurposing. Nat Rev Genet. 2021;22(10):658–71. doi: 10.1038/s41576-021-00387-z 34302145

[21] Pushpakom S, Iorio F, Eyers PA, Escott KJ, Hopper S, Wells A, et al. Drug repurposing: progress, challenges and recommendations. Nat Rev Drug Discov. 2019;18(1):41–58. doi: 10.1038/nrd.2018.168 30310233

[22] Zhang H, Fan C, Li L, Liu F, Li S, Ma L, et al. Repurposing the memory-promoting meclofenoxate hydrochloride as a treatment for Parkinson’s disease through integrative multi-omics analysis. NPJ Parkinsons Dis. 2025;11(1):167. doi: 10.1038/s41531-025-01027-7 40514362 PMC12166093

[23] Fang J, Zhang P, Wang Q, Chiang C-W, Zhou Y, Hou Y, et al. Artificial intelligence framework identifies candidate targets for drug repurposing in Alzheimer’s disease. Alzheimers Res Ther. 2022;14(1):7. doi: 10.1186/s13195-021-00951-z 35012639 PMC8751379

[24] Langfelder P, Horvath S. WGCNA: an R package for weighted correlation network analysis. BMC Bioinformatics. 2008;9:559. doi: 10.1186/1471-2105-9-559 19114008 PMC2631488

[25] Tesson BM, Breitling R, Jansen RC. DiffCoEx: a simple and sensitive method to find differentially coexpressed gene modules. BMC Bioinformatics. 2010;11:497. doi: 10.1186/1471-2105-11-497 20925918 PMC2976757

[26] Cabili MN, Trapnell C, Goff L, Koziol M, Tazon-Vega B, Regev A, et al. Integrative annotation of human large intergenic noncoding RNAs reveals global properties and specific subclasses. Genes Dev. 2011;25(18):1915–27. doi: 10.1101/gad.17446611 21890647 PMC3185964

[27] Skene NG, Bryois J, Bakken TE, Breen G, Crowley JJ, Gaspar HA, et al. Genetic identification of brain cell types underlying schizophrenia. Nat Genet. 2018;50(6):825–33. doi: 10.1038/s41588-018-0129-5 29785013 PMC6477180

[28] Wang D, Liu S, Warrell J, Won H, Shi X, Navarro FCP, et al. Comprehensive functional genomic resource and integrative model for the human brain. Science. 2018;362(6420):eaat8464. doi: 10.1126/science.aat8464 30545857 PMC6413328

[29] Gandal MJ, Zhang P, Hadjimichael E, Walker RL, Chen C, Liu S, et al. Transcriptome-wide isoform-level dysregulation in ASD, schizophrenia, and bipolar disorder. Science. 2018;362(6420):eaat8127. doi: 10.1126/science.aat8127 30545856 PMC6443102

[30] Sey NYA, Hu B, Mah W, Fauni H, McAfee JC, Rajarajan P, et al. A computational tool (H-MAGMA) for improved prediction of brain-disorder risk genes by incorporating brain chromatin interaction profiles. Nat Neurosci. 2020;23(4):583–93. doi: 10.1038/s41593-020-0603-0 32152537 PMC7131892

[31] Trubetskoy V, Pardiñas AF, Qi T, Panagiotaropoulou G, Awasthi S, Bigdeli TB, et al. Mapping genomic loci implicates genes and synaptic biology in schizophrenia. Nature. 2022;604(7906):502–8. doi: 10.1038/s41586-022-04434-5 35396580 PMC9392466

[32] Trabzuni D, Ramasamy A, Imran S, Walker R, Smith C, Weale ME, et al. Widespread sex differences in gene expression and splicing in the adult human brain. Nat Commun. 2013;4:2771. doi: 10.1038/ncomms3771 24264146 PMC3868224

[33] Ramasamy A, Trabzuni D, Guelfi S, Varghese V, Smith C, Walker R, et al. Genetic variability in the regulation of gene expression in ten regions of the human brain. Nat Neurosci. 2014;17(10):1418–28. doi: 10.1038/nn.3801 25174004 PMC4208299

[34] Trabzuni D, Ryten M, Emmett W, Ramasamy A, Lackner KJ, Zeller T, et al. Fine-mapping, gene expression and splicing analysis of the disease associated LRRK2 locus. PLoS One. 2013;8(8):e70724. doi: 10.1371/journal.pone.0070724 23967090 PMC3742662

[35] Pirker W, Katzenschlager R, Hallett M, Poewe W. Pharmacological treatment of tremor in Parkinson’s disease revisited. J Parkinsons Dis. 2023;13(2):127–44. 36847017 10.3233/JPD-225060 PMC10041452

[36] Carpentier AF, Bonnet AM, Vidailhet M, Agid Y. Improvement of levodopa-induced dyskinesia by propranolol in Parkinson’s disease. Neurology. 1996;46(6):1548–51. doi: 10.1212/wnl.46.6.1548 8649546

[37] Fray MJ, Dickinson RP. Discovery of potent and selective succinyl hydroxamate inhibitors of matrix metalloprotease-3 (stromelysin-1). Bioorg Med Chem Lett. 2001;11(4):571–4. doi: 10.1016/s0960-894x(00)00720-4 11229774

[38] Poewe W, Seppi K, Tanner CM, Halliday GM, Brundin P, Volkmann J, et al. Parkinson disease. Nat Rev Dis Primers. 2017;3:17013. doi: 10.1038/nrdp.2017.13 28332488

[39] Kukkle PL, Kaladiyil AP, Geetha TS, Menon R, Mridula Kandadai R, Goyal V. Association of Bassoon (BSN) Gene Mutations with Gait and Motor Impairments in Parkinson’s Disease. medRxiv. 2025. doi: 2025.08.10.25333397 10.1111/ejn.70458 41802976

[40] Fornito A, Zalesky A, Breakspear M. The connectomics of brain disorders. Nat Rev Neurosci. 2015;16(3):159–72. doi: 10.1038/nrn3901 25697159

[41] Kaufmann T, van der Meer D, Doan NT, Schwarz E, Lund MJ, Agartz I, et al. Common brain disorders are associated with heritable patterns of apparent aging of the brain. Nat Neurosci. 2019;22(10):1617–23. doi: 10.1038/s41593-019-0471-7 31551603 PMC6823048

[42] Crossley NA, Mechelli A, Scott J, Carletti F, Fox PT, McGuire P, et al. The hubs of the human connectome are generally implicated in the anatomy of brain disorders. Brain. 2014;137(Pt 8):2382–95. doi: 10.1093/brain/awu132 25057133 PMC4107735

[43] Hu H-Y, Hu H, Jiang J, Bi Y-L, Sun Y, Ou Y-N, et al. Echocardiographic measures of the left heart and cerebrospinal fluid biomarkers of Alzheimer’s disease pathology in cognitively intact adults: The CABLE study. Alzheimers Dement. 2024;20(6):3943–57. doi: 10.1002/alz.13837 38676443 PMC11180853

[44] Chen P-H, Hsiao C-Y, Chiang S-J, Chung K-H, Tsai S-Y. Association of lipids and inflammatory markers with left ventricular wall thickness in patients with bipolar disorder. J Affect Disord. 2024;358:12–8. doi: 10.1016/j.jad.2024.05.020 38705523

[45] Davis KL, Davis BM, Greenwald BS, Mohs RC, Mathé AA, Johns CA, et al. Cortisol and Alzheimer’s Disease, I: Basal Studies. Am J Psychiatry. 1986. 10.1176/ajp.143.3.300 3953862

[46] Csernansky JG, Dong H, Fagan AM, Wang L, Xiong C, Holtzman DM, et al. Plasma cortisol and progression of dementia in DAT subjects. Am J Psychiatry. 2006. 10.1176/appi.ajp.163.12.2164 PMC1780275 17151169

[47] Milligan Armstrong A, Porter T, Quek H, White A, Haynes J, Jackaman C, et al. Chronic stress and Alzheimer’s disease: the interplay between the hypothalamic-pituitary-adrenal axis, genetics and microglia. Biol Rev Camb Philos Soc. 2021;96(5):2209–28. doi: 10.1111/brv.12750 34159699

[48] Walker E, Mittal V, Tessner K. Stress and the hypothalamic pituitary adrenal axis in the developmental course of schizophrenia. Annu Rev Clin Psychol. 2008;4:189–216. doi: 10.1146/annurev.clinpsy.4.022007.141248 18370616

[49] Purves-Tyson TD, Weber-Stadlbauer U, Richetto J, Rothmond DA, Labouesse MA, Polesel M, et al. Increased levels of midbrain immune-related transcripts in schizophrenia and in murine offspring after maternal immune activation. Mol Psychiatry. 2021;26(3):849–63. doi: 10.1038/s41380-019-0434-0 31168068 PMC7910216

[50] Howes OD, Williams M, Ibrahim K, Leung G, Egerton A, McGuire PK, et al. Midbrain dopamine function in schizophrenia and depression: a post-mortem and positron emission tomographic imaging study. Brain. 2013;136(Pt 11):3242–51. doi: 10.1093/brain/awt264 24097339 PMC3808688

[51] Zhu Y, Owens SJ, Murphy CE, Ajulu K, Rothmond D, Purves-Tyson T, et al. Inflammation-related transcripts define “high” and “low” subgroups of individuals with schizophrenia and bipolar disorder in the midbrain. Brain Behav Immun. 2022;105:149–59. doi: 10.1016/j.bbi.2022.06.012 35764269

[52] D’Amelio M, Puglisi-Allegra S, Mercuri N. The role of dopaminergic midbrain in Alzheimer’s disease: translating basic science into clinical practice. Pharmacol Res. 2018;130:414–9. doi: 10.1016/j.phrs.2018.01.016 29391234

[53] Hassan A, Benarroch EE. Heterogeneity of the midbrain dopamine system. Neurology. 2015;85(20):1795–805. doi: 10.1212/wnl.0000000000002137 26475693

[54] Smajić S, Prada-Medina CA, Landoulsi Z, Ghelfi J, Delcambre S, Dietrich C, et al. Single-cell sequencing of human midbrain reveals glial activation and a Parkinson-specific neuronal state. Brain. 2022;145(3):964–78. doi: 10.1093/brain/awab446 34919646 PMC9050543

[55] Chung S, Lee EJ, Yun S, Choe HK, Park S-B, Son HJ, et al. Impact of circadian nuclear receptor REV-ERBα on midbrain dopamine production and mood regulation. Cell. 2014;157(4):858–68. doi: 10.1016/j.cell.2014.03.039 24813609

[56] Kim YS, Kim SS, Cho JJ, Choi DH, Hwang O, Shin DH, et al. Matrix metalloproteinase-3: a novel signaling proteinase from apoptotic neuronal cells that activates microglia. J Neurosci. 2005;25(14):3701–11. doi: 10.1523/JNEUROSCI.4346-04.2005 15814801 PMC6725382

[57] Kim ST, Kim E-M, Choi JH, Son HJ, Ji IJ, Joh TH, et al. Matrix metalloproteinase-3 contributes to vulnerability of the nigral dopaminergic neurons. Neurochem Int. 2010;56(1):161–7. doi: 10.1016/j.neuint.2009.09.014 19815046

[58] Walker EJ, Rosenberg GA. TIMP-3 and MMP-3 contribute to delayed inflammation and hippocampal neuronal death following global ischemia. Exp Neurol. 2009;216(1):122–31. doi: 10.1016/j.expneurol.2008.11.022 19111539 PMC2709713

[59] Kim E-M, Hwang O. Role of matrix metalloproteinase-3 in neurodegeneration. J Neurochem. 2011;116(1):22–32. doi: 10.1111/j.1471-4159.2010.07082.x 21044079

[60] Wang X-X, Tan M-S, Yu J-T, Tan L. Matrix metalloproteinases and their multiple roles in Alzheimer’s disease. Biomed Res Int. 2014;2014:908636. doi: 10.1155/2014/908636 25050378 PMC4094696

[61] Waites CL, Leal-Ortiz SA, Okerlund N, Dalke H, Fejtova A, Altrock WD, et al. Bassoon and Piccolo maintain synapse integrity by regulating protein ubiquitination and degradation. EMBO J. 2013;32(7):954–69. doi: 10.1038/emboj.2013.27 23403927 PMC3616282

[62] Ivanova D, Dirks A, Fejtova A. Bassoon and piccolo regulate ubiquitination and link presynaptic molecular dynamics with activity-regulated gene expression. J Physiol. 2016;594(19):5441–8. doi: 10.1113/JP271826 26915533 PMC5043050

[63] Montenegro-Venegas C, Guhathakurta D, Pina-Fernandez E, Andres-Alonso M, Plattner F, Gundelfinger ED, et al. Bassoon controls synaptic vesicle release via regulation of presynaptic phosphorylation and cAMP. EMBO Rep. 2022;23(8):e53659. doi: 10.15252/embr.202153659 35766170 PMC9346490

[64] Mendoza Schulz A, Jing Z, Sánchez Caro JM, Wetzel F, Dresbach T, Strenzke N, et al. Bassoon-disruption slows vesicle replenishment and induces homeostatic plasticity at a CNS synapse. EMBO J. 2014;33(5):512–27. doi: 10.1002/embj.201385887 24442636 PMC3989631

[65] He D, Fan C, Qi M, Yang Y, Cooper DN, Zhao H. Prioritization of schizophrenia risk genes from GWAS results by integrating multi-omics data. Transl Psychiatry. 2021;11(1):175. doi: 10.1038/s41398-021-01294-x 33731678 PMC7969765

[66] Zhang T, Zhang S-W, Xie M-Y, Li Y. A novel heterophilic graph diffusion convolutional network for identifying cancer driver genes. Brief Bioinform. 2023;24(3):bbad137. doi: 10.1093/bib/bbad137 37055234

[67] Darmanis S, Sloan SA, Zhang Y, Enge M, Caneda C, Shuer LM, et al. A survey of human brain transcriptome diversity at the single cell level. Proc Natl Acad Sci U S A. 2015;112(23):7285–90. doi: 10.1073/pnas.1507125112 26060301 PMC4466750

[68] Badanjak K, Mulica P, Smajic S, Delcambre S, Tranchevent L-C, Diederich N, et al. iPSC-derived microglia as a model to study inflammation in idiopathic Parkinson’s Disease. Front Cell Dev Biol. 2021;9:740758. doi: 10.3389/fcell.2021.740758 34805149 PMC8602578

[69] Aleknonytė-Resch M, Trinh J, Leonard H, Delcambre S, Leitão E, Lai D, et al. Genome-wide case-only analysis of gene-gene interactions with known Parkinson’s disease risk variants reveals link between LRRK2 and SYT10. NPJ Parkinsons Dis. 2023;9(1):102. doi: 10.1038/s41531-023-00550-9 37386035 PMC10310744

[70] Kang HJ, Kawasawa YI, Cheng F, Zhu Y, Xu X, Li M, et al. Spatio-temporal transcriptome of the human brain. Nature. 2011;478(7370):483–9. doi: 10.1038/nature10523 22031440 PMC3566780

[71] Huo Q, Chen M, He Q, Zhang J, Li B, Jin K, et al. Prefrontal cortical GABAergic dysfunction contributes to aberrant UP-State duration in APP knockout mice. Cereb Cortex. 2017;27(8):4060–72. doi: 10.1093/cercor/bhw218 27552836

[72] Willsey AJ, Sanders SJ, Li M, Dong S, Tebbenkamp AT, Muhle RA, et al. Coexpression networks implicate human midfetal deep cortical projection neurons in the pathogenesis of autism. Cell. 2013;155(5):997–1007. doi: 10.1016/j.cell.2013.10.020 24267886 PMC3995413

[73] Velmeshev D, Perez Y, Yan Z, Valencia JE, Castaneda-Castellanos DR, Wang L, et al. Single-cell analysis of prenatal and postnatal human cortical development. Science. 2023;382(6667):eadf0834. doi: 10.1126/science.adf0834 37824647 PMC11005279

[74] Moran LB, Duke DC, Deprez M, Dexter DT, Pearce RKB, Graeber MB. Whole genome expression profiling of the medial and lateral substantia nigra in Parkinson’s disease. Neurogenetics. 2006;7(1):1–11. doi: 10.1007/s10048-005-0020-2 16344956

[75] Duke DC, Moran LB, Pearce RKB, Graeber MB. The medial and lateral substantia nigra in Parkinson’s disease: mRNA profiles associated with higher brain tissue vulnerability. Neurogenetics. 2007;8(2):83–94. doi: 10.1007/s10048-006-0077-6 17211632

[76] Nalls MA, Blauwendraat C, Vallerga CL, Heilbron K, Bandres-Ciga S, Chang D, et al. Identification of novel risk loci, causal insights, and heritable risk for Parkinson’s disease: a meta-analysis of genome-wide association studies. Lancet Neurol. 2019;18(12):1091–102. doi: 10.1016/S1474-4422(19)30320-5 31701892 PMC8422160

[77] Lee C, Kim J, Shin SG, Hwang S. Absolute and relative QPCR quantification of plasmid copy number in Escherichia coli. J Biotechnol. 2006;123(3):273–80. doi: 10.1016/j.jbiotec.2005.11.014 16388869

[78] Lesnick TG, Papapetropoulos S, Mash DC, Ffrench-Mullen J, Shehadeh L, de Andrade M, et al. A genomic pathway approach to a complex disease: axon guidance and Parkinson disease. PLoS Genet. 2007;3(6):e98. doi: 10.1371/journal.pgen.0030098 17571925 PMC1904362

[79] Patil I. Visualizations with statistical details: The “ggstatsplot” approach. JOSS. 2021;6(61):3167. doi: 10.21105/joss.03167

