# Supplementary material for: A prototype-augmented graph representation learning framework for identifying brain disorder-associated genes and facilitating drug repurposing
Source: PLoS Comput Biol. 2026 May 29;22(5):e1014323. doi: 10.1371/journal.pcbi.1014323 (PMC13221074; doi:10.1371/journal.pcbi.1014323)
Supplement: S1 Text — (DOCX) [file pcbi.1014323.s001.docx]

**Supplementary Information For**

**A** **prototype-augmented graph representation learning framework for identifying brain disorder-associated genes and facilitating drug repurposing**

**Supplementary Methods**

**Collecting SNPs associated with disorders**

The SNPs significantly associated with SCZ were obtained from a two-stage genome-wide association study of up to 76,755 individuals with schizophrenia and 243,649 control individuals[[1](#_ENREF_1)]. For the SNPs significantly associated with AD, we downloaded from four sources, including three previous studies[[2-4](#_ENREF_2)]and the GWAS catalog[[5](#_ENREF_5)]. For the other three disorders, we extracted SNPs by following the procedures described in a previous study[[6](#_ENREF_6)] from the GWAS catalog[[5](#_ENREF_5)]. The details about the SNP-associated five brain disorders were listed in S13_Table.

**Multi-omics data used in the MOGT**

We collected DE and EPI data according to the previous research process[[6](#_ENREF_6)]. DE is the significance level-value of genes expressed differentially in patients and controls by analysis of RNA-seq data from the samples listed in S17_Table. EPI data were collected from two studies. The first study constructed Hi-C libraries from two major zones: the cortical and subcortical plate (CP) and the germinal zone (GZ)[[7](#_ENREF_7)]. The second study described enhancer-promoter interactions in different cell types from cortical brain tissue from 10 individuals, including microglia (60,162 interactions), neurons (62,419 interactions), and oligodendrocytes (41,069 interactions)(S18_Table)[[8](#_ENREF_8)]. We downloaded RNA sequencing data on the developmental stage specificity of expression of genes in brain tissues from BrainSpan (<https://www.brainspan.org>), including five brain regions (Parietal Lobe, Frontal Lobe, Temporal Lobe, Cerebellum, Occipital Lobe) in two developmental stages (adolescent and adult).

**Drug candidate discovery**

We performed drug candidate discovery as previously reported[[9](#_ENREF_9)]. We generated consensus co-expression modules (CCMs) common to all ten brain regions by WGCNA[[10](#_ENREF_10)]. The parameters were set as follows: β = 7 (chosen based on the scale-free topology criterion r^2^ > 0.8), minModuleSize = 30, mergeCutHeight = 0.25, maxBlockSize = 6000, and corType = 'cor', which means calculating the Pearson correlation factor of the similarity between genes. We first generated a separate topological overlap matrix (TOM) for each brain region. The component-wise minimum of the TOMs in each brain region was then extracted to generate a consensus TOM. The clustering was done using the function hclust with parameter method = "average", which is a standard hierarchical clustering with average linkage. The consensus co-expression modules were defined as branches of the clustering tree extracted by the function cutreeDynamic.

Specific co-expressed modules (SCMs) represented gene modules specifically expressed in each of the ten brain regions compared to the other nine brain regions, which were generated by Diffcoex[[11](#_ENREF_11)]. For a given condition b, we define the adjacency matrix $A^{b}$ as the correlation for all pairs of genes $\left( i,j \right)$:

*$A^{b}:a_{ij}^{b} = cor(\text{gene}_{i},\text{gene}_{j})$*,

The correlation measure cor could be a Pearson or a Spearman coefficient. For the adjacency matrix of a given brain region $C^{o}$ and the other nine brain regions $C^{n}$, the matrix of adjacency difference is given by:

**,

The soft threshold parameter $\beta\left( \beta>0 \right)$ is used to transform the correlation values. It gives higher weight to larger correlation differences than to smaller, less meaningful differences. The adjacent difference matrix D is used as input for clustering, and modules are identified. The clustering was done using the function hclust with parameter method = "average". We then used cutreeDynamic to extract modules from the resulting dendrogram. The parameters were set as follows: method="hybrid", cutHeight=0.996, minClusterSize = 30.

**Supplementary** **Results**

**Validation of GSK-1059615 in PD model**

GSK-1059615 is a dual inhibitor of PI3Kα/β/δ/γ (reversible) and mTOR with IC50 of 0.4 nM, 0.6 nM, 2 nM, 5 nM, and 12 nM, respectively[[12](#_ENREF_12)]. Previous studies have demonstrated that the PI3K/Akt/mTOR pathway exerts cell-type-specific effects in Parkinson’s disease: its activation in neurons partially reverses pathological phenotypes[[13](#_ENREF_13), [14](#_ENREF_14)], whereas in glial cells it exacerbates neuroinflammation[[15-17](#_ENREF_15)]. Accordingly, we inhibited this pathway in primary astrocytes using GSK-1059615 to see if it could reduce the inflammatory profile. First, a PD-relevant astrocytic model was established by rotenone exposure (20, 10, 5 μM/ml for 24 hours; 20, 15, 10, 5 μM/ml for 48 hours) (Fig. S8e, f), and the cytotoxic effects of GSK-1059615 were assessed. At high concentrations (2000, 1000, 500, 200 nM/ml), GSK-1059615 exhibited pronounced toxicity (Fig. S8g). Guided by prior reports[[12](#_ENREF_12)], we selected a descending dose series of 24, 12, and 6 nM/ml for further testing. CCK-8 assays showed no significant improvement in cell viability at 24 or 12 nM/ml (Fig. S8h); at 6 nM/ml, a slight decrease in viability was observed. Subsequent Calcein-AM/PI staining confirmed statistically significant effects on cell viability but had a relatively minor biological impact on viability (Fig. S8i, j). Consequently, GSK-1059615 was not included in further experiments.

**References**

1. Trubetskoy V, Pardinas AF, Qi T, Panagiotaropoulou G, Awasthi S, Bigdeli TB, et al. Mapping genomic loci implicates genes and synaptic biology in schizophrenia. Nature. 2022;604(7906):502-8.

2. Wightman DP, Jansen IE, Savage JE, Shadrin AA, Bahrami S, Holland D, et al. A genome-wide association study with 1,126,563 individuals identifies new risk loci for Alzheimer's disease. Nat Genet. 2021;53(9):1276-82.

3. Andrews SJ, Fulton-Howard B, Goate A. Interpretation of risk loci from genome-wide association studies of Alzheimer's disease. Lancet Neurol. 2020;19(4):326-35.

4. Bellenguez C, Kucukali F, Jansen IE, Kleineidam L, Moreno-Grau S, Amin N, et al. New insights into the genetic etiology of Alzheimer's disease and related dementias. Nat Genet. 2022;54(4):412-36.

5. Buniello A, MacArthur JAL, Cerezo M, Harris LW, Hayhurst J, Malangone C, et al. The NHGRI-EBI GWAS Catalog of published genome-wide association studies, targeted arrays and summary statistics 2019. Nucleic Acids Res. 2019;47(D1):D1005-D12.

6. He D, Li L, Zhang H, Liu F, Li S, Xiu X, et al. Accurate identification of genes associated with brain disorders by integrating heterogeneous genomic data into a Bayesian framework. EBioMedicine. 2024;107:105286.

7. Won H, de la Torre-Ubieta L, Stein JL, Parikshak NN, Huang J, Opland CK, et al. Chromosome conformation elucidates regulatory relationships in developing human brain. Nature. 2016;538(7626):523-7.

8. Nott A, Holtman IR, Coufal NG, Schlachetzki JCM, Yu M, Hu R, et al. Brain cell type-specific enhancer-promoter interactome maps and disease-risk association. Science. 2019;366(6469):1134-9.

9. Zhang H, Fan C, Li L, Liu F, Li S, Ma L, et al. Repurposing the memory-promoting meclofenoxate hydrochloride as a treatment for Parkinson's disease through integrative multi-omics analysis. NPJ Parkinsons Dis. 2025;11(1):167.

10. Langfelder P, Horvath S. WGCNA: an R package for weighted correlation network analysis. BMC Bioinformatics. 2008;9:559.

11. Tesson BM, Breitling R, Jansen RC. DiffCoEx: a simple and sensitive method to find differentially coexpressed gene modules. BMC Bioinformatics. 2010;11:497.

12. Carnero A. Novel inhibitors of the PI3K family. Expert Opin Investig Drugs. 2009;18(9):1265-77.

13. Nakano N, Matsuda S, Ichimura M, Minami A, Ogino M, Murai T, et al. PI3K/AKT signaling mediated by G protein‑coupled receptors is involved in neurodegenerative Parkinson's disease (Review). Int J Mol Med. 2017;39(2):253-60.

14. Wang L, Tian S, Ruan S, Wei J, Wei S, Chen W, et al. Neuroprotective effects of cordycepin on MPTP-induced Parkinson's disease mice via suppressing PI3K/AKT/mTOR and MAPK-mediated neuroinflammation. Free Radic Biol Med. 2024;216:60-77.

15. Liu K, An J, Zhang J, Zhao J, Sun P, He Z. Network pharmacology combined with experimental validation show that apigenin as the active ingredient of Campsis grandiflora flower against Parkinson's disease by inhibiting the PI3K/AKT/NF-κB pathway. PLoS One. 2024;19(10):e0311824.

16. Chung CY, Singh K, Sheshadri P, Valdebenito GE, Chacko AR, Costa Besada MA, et al. Inhibition of the PI3K-AKT-MTORC1 axis reduces the burden of the m.3243A>G mtDNA mutation by promoting mitophagy and improving mitochondrial function. Autophagy. 2025;21(4):881-96.

17. Zhuo Y, Li WS, Lu W, Li X, Ge LT, Huang Y, et al. TGF-β1 mediates hypoxia-preconditioned olfactory mucosa mesenchymal stem cells improved neural functional recovery in Parkinson's disease models and patients. Mil Med Res. 2024;11(1):48.
